# Shell Thickness and Heterogeneity Dependence of Triplet Energy Transfer between Core–Shell Quantum Dots and Adsorbed Molecules

**DOI:** 10.1021/jacs.5c01838

**Published:** 2025-04-30

**Authors:** Tao Jin, Zhendian Zhang, Sheng He, Alexey L. Kaledin, Zihao Xu, Yawei Liu, Peng Zhang, David N. Beratan, Tianquan Lian

**Affiliations:** † Department of Chemistry, Emory University, 1515 Dickey Dr, Atlanta, Georgia 30322, United States; ‡ Department of Chemistry, Duke University, Durham, North Carolina 27708, United States; § Cherry L. Emerson Center for Scientific Computation, Emory University, 1515 Dickey Dr, Atlanta, Georgia 30322, United States; ∥ Department of Physics, Duke University, Durham, North Carolina 27708, United States; ⊥ Department of Biochemistry, Duke University, Durham, North Carolina 27710, United States

## Abstract

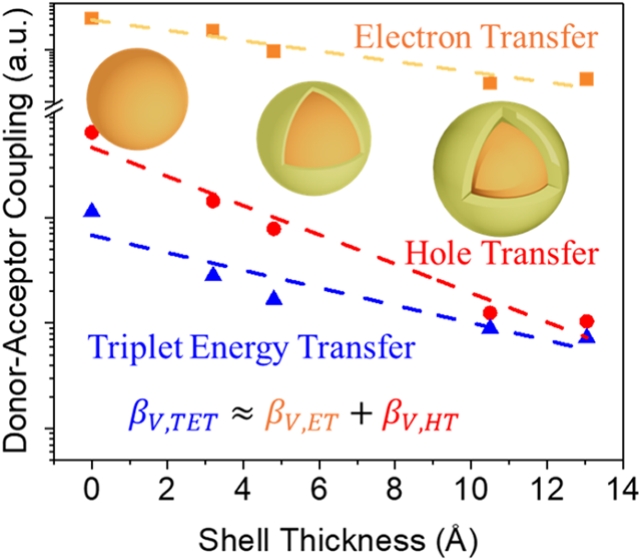

Quantum dot (QD)-sensitized triplet energy transfer (TET) has found promising applications in photon upconversion and photocatalysis. However, the underlying mechanism of TET in the QD-acceptor complex remains unclear despite the well-developed TET theory for the molecular donor–acceptor systems. Herein, the coupling strength of TET from CdSe/CdS core–shell QDs to 9-anthracene carboxylic acid (ACA) was studied by measuring the TET rate as a function of shell thickness with time-resolved photoluminescence. The change of TET-coupling strength with increasing shell thickness was further compared to those of electron and hole transfers from QDs so that we could test whether QD-sensitized TET is mediated by the charge transfer virtual state and can be considered as simultaneous electron and hole transfers as in molecular donor–acceptor systems. The measured coupling strength of TET from the CdSe/CdS QD decreases exponentially with the CdS shell thickness *r*: |*V*|(*r*) = |*V*|(0)e^–βr^, with an exponential decay factor β of 0.19 Å^–1^, which is smaller than the sum of the measured decay factors for electron transfer to methyl viologen (0.18 Å^–1^) and hole transfer to phenothiazine (0.29 Å^–1^) from the same QD. This inconsistency is explained by the broadening of QD shell thicknesses in the distance dependence study, which significantly modifies the TET-coupling strength and driving force, resulting in a shallower distance dependence of the TET rate constants. This study sheds light on the fundamental mechanisms of QD-sensitized TET reactions.

## Introduction

Efficient generation of long-lived triplet excited states has been extensively studied during the past decades due to its promising applications in photon upconversion,^1−5^ photodynamic therapy,^6−8^ and photocatalysis.^9−11^ Traditional methods of triplet excited state generation in molecules include intersystem crossing and triplet energy transfer (TET) from molecular sensitizers,^4,12,13^ both of which require intersystem crossing in molecules and suffer from large energy loss due to the large singlet–triplet energy gap in molecules. Recently developed quantum dot (QD)-sensitized TET has received intense research interest^14−18^ because it utilizes the unique properties of QDs, including the small singlet–triplet energy gap,^19−21^ broad absorption spectral range,^22,23^ large extinction coefficient,^23,24^ and tunability in structures and photophysical properties.^25−27^ TET sensitized by CdSe,^14,28−30^ CdS,^31^ PbS,^32,33^ and perovskite QDs^34,35^ has been successfully developed and incorporated in photon upconversion and photocatalysis.^36−39^ Progress has also been made in improving TET efficiency through optimizing QD quantum yield,^40^ passivating QD surface,^31,41^ suppressing side reaction pathways,^32^ and modifying surface ligands.^42^

TET in molecular donor–acceptor systems is well described by Dexter Energy Transfer (DET) involving the simultaneous transfers of an electron and hole.^43−46^ The original DET theory was based on the Fermi Golden rule with coupling strength given by a two-electron exchange integral, which scales with wave function overlap and decays exponentially with the distance between the donor and acceptor.^43,47^ Later research of DET coupling strength emphasized the contribution of one-electron integral terms of TET mediated by charge transfer virtual states.^48,49^ Recently, higher energy virtual states including bridge exciton states were proposed to be significant in donor-bridge-acceptor systems with low-bridge tunneling barrier and long bridge length.^50,51^

Despite the development of DET theory in molecular donor–acceptor systems, whether the theory can be applied to QD-sensitized TET systems is still ambiguous. Specifically, the relative contribution of the two-electron exchange integral and the charge transfer virtual state is unclear in QD-sensitized TET. Time-resolved techniques, including transient absorption spectroscopy (TA) and time-resolved photoluminescence (TRPL), have been applied to probe the dynamics of TET, from which direct DET from band edge excitons has been proposed for most QD-acceptor complexes,^14,34,52^ and TET through charge separated states and trap state intermediates has also been identified for some systems.^53−55^ However, the Dexter energy transfer mechanism reported in previous studies was inferred from the lack of charge transfer evidence or the concomitant growth kinetics of the acceptor triplet and the decay kinetics of the QD exciton state rather than from examining the coupling of the energy transfer and electron and hole transfers.

As the critical factor to determine the TET rate, the coupling strength of TET has been studied for QD-acceptor systems.^34,42,56^ The coupling strength can be tuned by varying the QD size,^34^ QD ligand length,^42^ the molecular spacer length,^56−58^ or inorganic shell thickness^30,31^ between the QD and the acceptor, and it has been demonstrated that the upconversion yield and TET rate decrease with reduced coupling strength in complexes of perovskite-pyrene carboxylic acid,^34^ CdSe-phenyl bridge-anthracene,^56^ CdSe-oligoyne bridge-anthracene,^58^ PbS-phenyl bridge-tetracene,^57^ PbS/CdS-rubrene,^41^ and CdSe/Zn(Cd)S-anthracene.^59^ These studies mainly focused on the phenomenological correlation between the exciton wave function density and the TET efficiency, while a fundamental insight into the coupling compositions is still missing and rigorous tests of models for describing TET-coupling strength in QD-acceptor complexes are yet to be reported. Moreover, different from molecular donor–acceptor systems with precise energy levels and geometry, QD-molecule donor–acceptor systems may lack a well-defined coupling strength or free energy change due to the ubiquitous heterogeneous size distribution of the QDs. With a strong quantum confinement effect, the donor–acceptor coupling and QD exciton energy are expected to be sensitive to the QD size. It is unclear how such heterogeneity would impact the mechanism of the QD-sensitized TET.

**Scheme 1:**
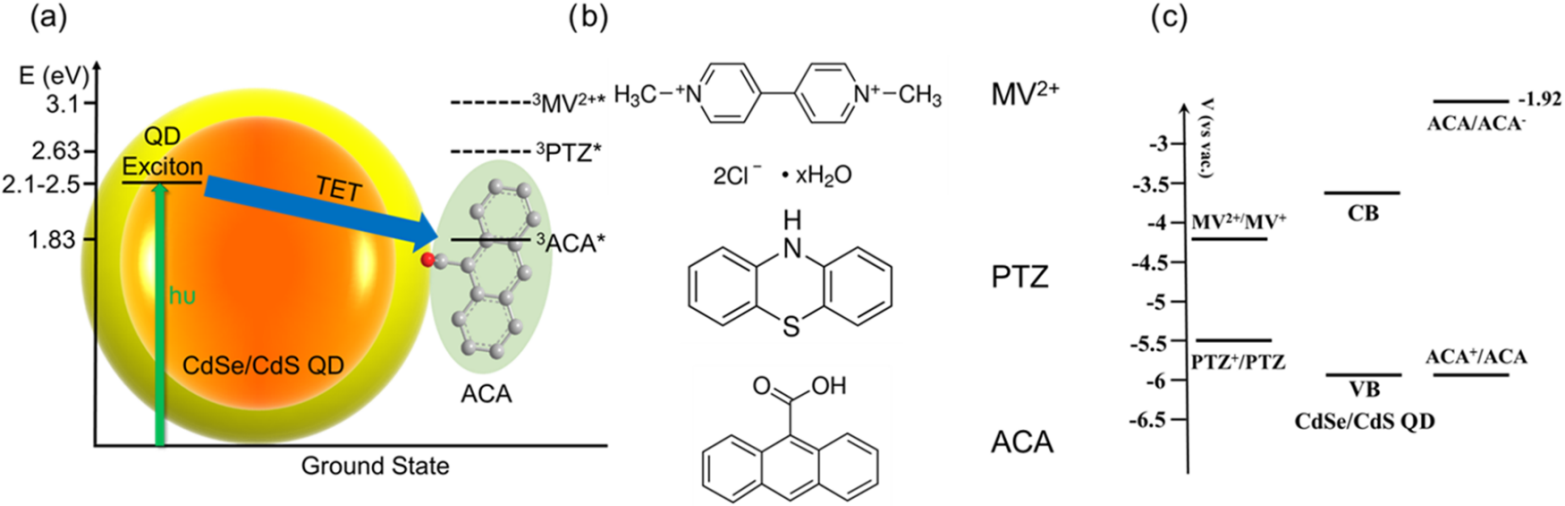
Energetics and Redox Potentials of the CdSe/CdS QDs, Triplet Energy Transfer (TET) Acceptor (ACA), Electron Transfer Acceptor (Methyl Viologen, MV^2+^), and Hole Transfer Acceptor (Phenothiazine, PTZ) in This Study (a) Scheme of TET from CdSe/CdS QDs to attached ACA acceptor. Energetics of QD, ^3^ACA*, ^3^MV^2+^*, and ^3^PTZ* are shown in the scheme.^61−63^ The dashed lines of ^3^MV^2+^* and ^3^PTZ* suggest energetically unfavorable TET from QD to MV^2+^ and PTZ, while the solid line of ^3^ACA* suggests energetically allowed TET from QD to ACA. (b) Molecular structures of MV^2+^, PTZ, and ACA. (c) Redox potentials of MV^2+^, PTZ, ACA, and conduction/valence band edge of CdSe/CdS QD.^60,63,64^

In this study, we aim to test whether the TET-coupling strength can be modeled with a scheme involving charge transfer virtual state; in other words, whether TET can be considered as simultaneous electron and hole transfers in QD-acceptor. CdSe/CdS core/shell QDs of varying shell thicknesses were used to systematically vary the coupling strength of TET from the QD to adsorbed acceptors. The dependence of TET-coupling strength on shell thickness was measured and compared to shell thickness dependence of electron and hole transfer coupling strength, with anthracene carboxylic acid (ACA), methyl viologen (MV^2+^), and phenothiazine (PTZ) as TET, electron transfer, and hole transfer acceptors, respectively (Scheme 1).^27,60^ The experimental result shows a weaker shell thickness dependence of TET-coupling strength than that expected from the model of charge transfer virtual state-mediated TET. This result is explained by a model that accounts for the effect of the heterogeneous distribution of shell thickness on the TET-coupling strength and driving force.

## Results and Discussion

### Characterization of CdSe/CdS Core–Shell QDs

CdSe/CdS core–shell QDs with various shell thicknesses were synthesized following the procedures reported in previous literature.^65^ The size distributions of the QDs were measured from transmission electron microscopy (TEM) images, which are shown in Supporting Information (SI) SI2 and Figure S1. By fitting diameter histograms with Gaussian functions (shown in Figure S2), we determined the average total diameter of the core/shell QDs (standard deviation) to be 2.94 (0.49) nm, 3.58 (0.59) nm, 3.90 (0.60) nm, 5.04 (1.00) nm, and 5.55 (0.90) nm, corresponding to 0, 0.9, 1.4, 3.1, and 3.8 monolayers (ML) of CdS shell, respectively.^27^ These samples are termed CdSe/CdS(X ML), where X is the number of CdS monolayers.

**Figure 1:**
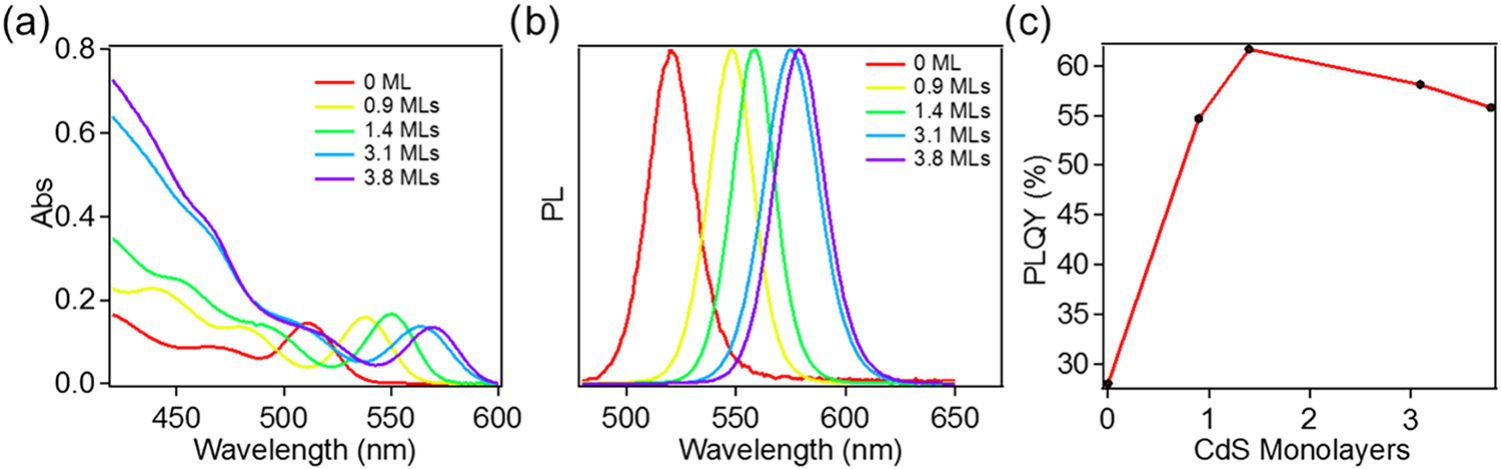
Absorption and emission spectra of the CdSe/CdS QDs. (a) UV–vis absorption spectra and (b) steady-state PL emission spectra of CdSe/CdS QDs with 0 (red), 0.9 (yellow), 1.4 (green), 3.1 (blue), and 3.8 (purple) monolayers of CdS shell. (c) PL quantum yields of CdSe/CdS QDs as a function of the CdS shell thickness.

Figure 1 shows the UV–vis absorption and photoluminescence (PL) spectra of the synthesized QDs. As shown in Figure 1a, with increasing shell thickness, the 1S_h_–1S_e_ absorption peak shifts from 511 to 570 nm, which can be attributed to the extension of the 1S_h_ and/or 1S_e_ wave functions into the CdS shell;^25^ the intensity of the bulk-like continuous absorption band (400–480 nm) corresponding to the transition between the higher hole levels and the 1S_e_ level (denoted as T band) increases with shell thickness.^27^ As shown in Figure 1b, the 1S exciton PL emission peak red shifts at larger CdS shell thicknesses from 521 nm for CdSe/CdS(0 ML) to 579 nm for CdSe/CdS(3.8 ML). The PL quantum yields (QYs) for the core–shell QDs increase from 28% in core-only QDs to 62% in CdSe/CdS(1.4 ML) (Figure 1c), because of the improved passivation of surface trap states of the CdSe core by the CdS shell. Further increase of shell thickness leads to slight decreases of the PL QY, which is likely caused by increased defects introduced in the synthesis procedure of further growth of the CdS shell.

Previous studies have shown that CdSe/CdS QDs of similar core size have a quasi-type II band alignment, in which the valence band edge of CdSe is higher than CdS and the conduction band edges degenerate throughout the core and shell.^27^ This band alignment can be confirmed by a transient absorption study (shown in SI3 and Figure S3). The excitation wavelength in the transient absorption study was set at 520 nm for all QD samples so that the 1S_h_–1S_e_ transition for CdSe/CdS(0/0.9/1.4 ML) or 2S_h_–1S_e_ transition for CdSe/CdS(3.1/3.8 ML) was excited. After the excitation, the 1S_h_–1S_e_ exciton was directly populated in CdSe/CdS(0/0.9/1.4 ML) and populated through hole relaxation from 2S_h_ to 1S_h_ for CdSe/CdS(3.1/3.8 ML). The population of 1S_h_–1S_e_ exciton results in bleaches at both 1S_h_–1S_e_ transition and T band, suggesting that the 1S electron wave function is delocalized in the entire QD, confirming the quasi-type II band alignment. TA studies show that with increasing shell thickness, the kinetics of 1S_h_–1S_e_ exciton bleach recovery (Figure S3f) become faster and more single exponential, which is attributed to the increasing probability of conduction band electron decay by radiative recombination with the valence band hole compared to the slow nonradiative recombination with trapped holes.^54^

### Core–Shell QD-Sensitized TET

**Figure 2:**
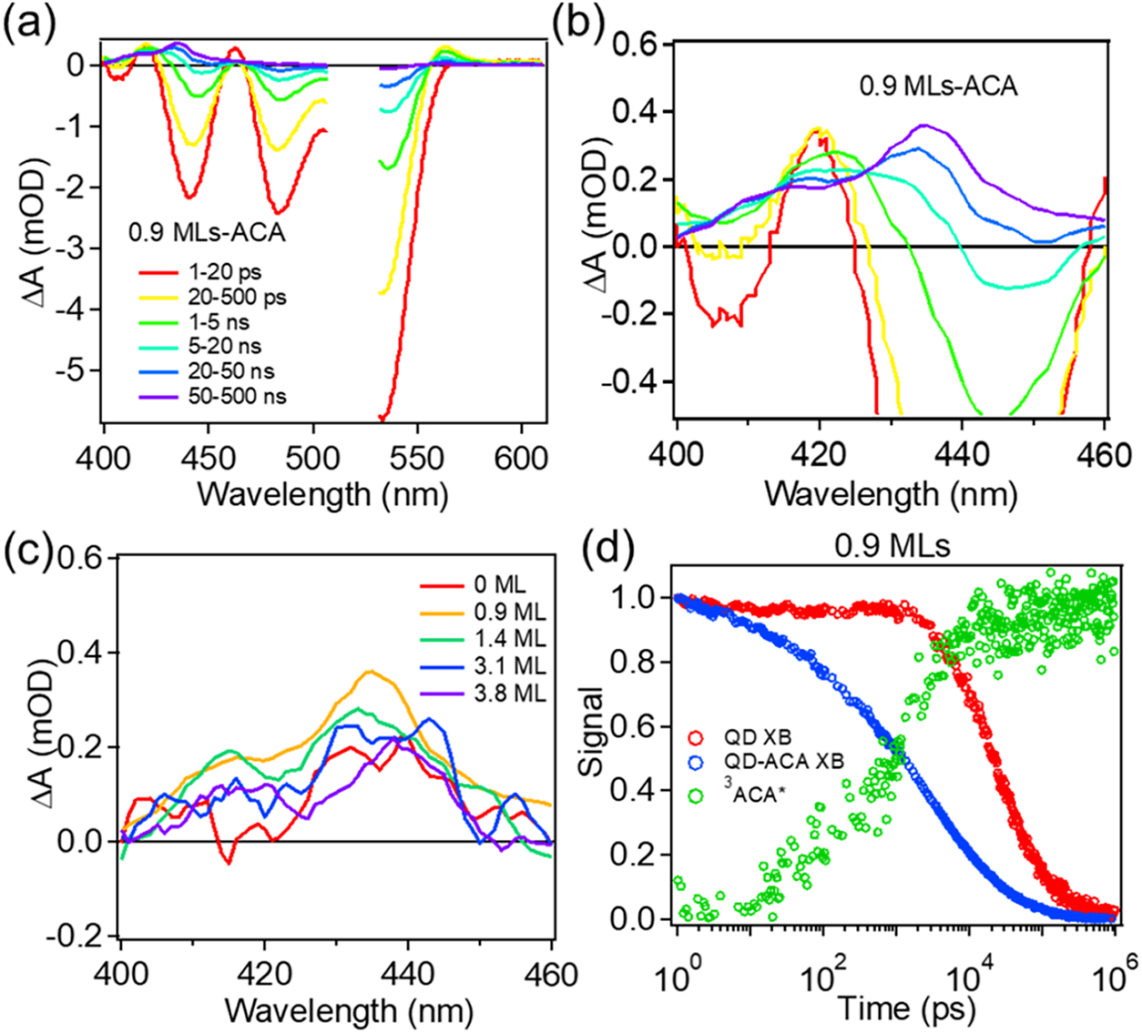
TA spectra and kinetics of QD-ACA. (a) Average TA spectra of CdSe/CdS(0.9 ML)-ACA measured under 520 nm excitation at indicated delay time windows. (b) Zoom-in figure of (a). (c) Average TA spectra of 50–500 ns in spectra range of ^3^ACA* signal (420–450 nm) for CdSe/CdS QD-ACA with 0 (red), 0.9 (orange), 1.4 (green), 3.1 (blue), and 3.8 (purple) MLs of CdS shell. (d) Normalized XB kinetics at the core 1S_h_–1S_e_ transition for QDs (red) and QD-ACA complex (blue) along with ^3^ACA* signal growth kinetics (green) in the time range of 1 ps–1 μs for CdSe/CdS(0.9 ML)-ACA. The ^3^ACA* growth kinetics were normalized to 1 at maximum for better comparison with QD-ACA XB decay.

TET from CdSe and CdSe/CdS QDs to 9-anthracene carboxylic acid (ACA) was studied with TA spectroscopy. A comparison of the absorption spectra of QD and QD-ACA complexes in Figure S4 shows that the adsorption of ACA has a negligible effect on the QD exciton band. TA measurements were conducted with 520 nm pump pulses, which selectively excite the QD exciton transition because of negligible absorptions of ACA at this wavelength. As shown in Figure 2 and Figure S5-1 in **SI5**, the QD-ACA TA spectra within 1 ns are dominated by QD features, including bleaches of both the 1S_h_–1S_e_ and T band transitions resulting from the state filling of the 1S electron level.^66,67^ The CdSe/CdS(0.9 ML)-ACA complex shows faster decay of the core exciton bleach (XB) signal compared to free QDs without ACA (Figure 2a). The faster XB decay is accompanied by the growth of ^3^ACA* T_1_→T_n_ signal from 420 to 450 nm (Figure 2b), suggesting TET from the CdSe/CdS QD to ACA.^14^ With an increasing CdS shell thickness, the ^3^ACA* signal growth can still be observed for all core–shell QDs. The signal amplitude of ^3^ACA* is larger for core–shell QDs with 0.9 monolayers of CdS than CdSe core-only QDs. However, the ^3^ACA* signal slightly decreases with a further increase of shell thickness (Figure 2c). The observation suggests the initial increase of TET efficiency from QDs after submonolayer shell growth and a slight decrease of TET efficiency with a further increase of shell thickness. Note that the TA spectra of QD-ACA show no features from the cation or anion radicals of ACA, indicating the one-step TET from QDs to ACA instead of sequential charge transfer to form ^3^ACA*.

The kinetics of QD XB (1S_h_–1S_e_ transition) and ^3^ACA* signal growth of QD-ACA complexes are compared in Figure 2d and Figure S5-2. The XB kinetics were directly obtained from kinetics at the corresponding XB peak position, while the ^3^ACA* signal was extracted from averaged kinetics from 430 to 435 nm after the subtraction of the scaled QD signal. As shown in Figure S5-2, the ^3^ACA* signal in CdSe QD-ACA does not grow until 1 ns, while there is a dramatic decrease of XB signal within 1 ns due to electron trapping.^54^ After 1 ns, the kinetics of ^3^ACA* growth matches the kinetics of XB decay of the QD-ACA complex, which suggests TET from QD to ACA. The growth of ^3^ACA* does not end until tens of nanoseconds. Compared to CdSe QD-ACA, CdSe/CdS QD-ACA complexes show a slight growth of ^3^ACA* within 1 ns, which ends mostly within 10 ns (Figure 2d). This suggests faster apparent TET rates for CdSe/CdS-ACA than for CdSe-ACA. For CdSe/CdS QD-ACA complexes, the XB decays faster than that of QDs without ACA due to TET, and the difference between the XB decays is smaller with increasing shell thickness, indicating a slower apparent TET rate. The result is consistent with the trend of the ^3^ACA* signal amplitude change in TA spectra, which suggests that the change of TET efficiencies in the studied QD-ACA complexes is mainly attributed to the change of TET apparent rates.

The apparent TET rate in QD-ACA complexes depends on the number of adsorbed ACA molecules per QD. Thus, in order to compare the TET-coupling strength in core–shell QD-ACA complexes with varying shell thicknesses, TET rates should be measured in complexes with the same number of adsorbed acceptors, which is difficult to do for the following reasons. In the TA experiment, an excess of ACA was added to a QD hexane solution for ultrasonication. Despite the relatively small solubility of ACA in hexane, it cannot be neglected when determining the number of adsorbed ACA on the QD surface from the UV–vis spectrum of QD-ACA. The ACA absorption in the UV–vis spectrum consists of contributions from both free ACA in solution and ACA attached on QD, and the determination of adsorbed ACA number on the QD surface from UV–vis spectra is not accurate. Furthermore, the XB kinetics in QDs do not accurately represent the band edge exciton population. For CdSe QDs, VB holes are trapped on the surface, and XB kinetics mostly represent the trap exciton dynamics.^54^ For CdSe/CdS QDs, surface trap states still exist, and their contribution to the XB kinetics cannot be neglected, considering the nonunity PL quantum yields of the QDs. Therefore, the QD XB decay and ^3^ACA* growth kinetics contain the contributions of band edge exciton and trap exciton populations, which complicate the analysis. It has been shown previously that in the CdSe-ACA complex, the TET rate from trap excitons is slower than that from band edge excitons and depends on the trap state energy.^54^ For the CdSe/CdS-ACA complex, the contribution of deep trap excitons to TET is unknown and is difficult to study due to a lack of spectral fingerprints of the deep trap excitons in both the photoinduced absorption signal in TA spectra and trap exciton emission in PL spectra.

### Shell Thickness Dependence of TET Rate

**Figure 3:**
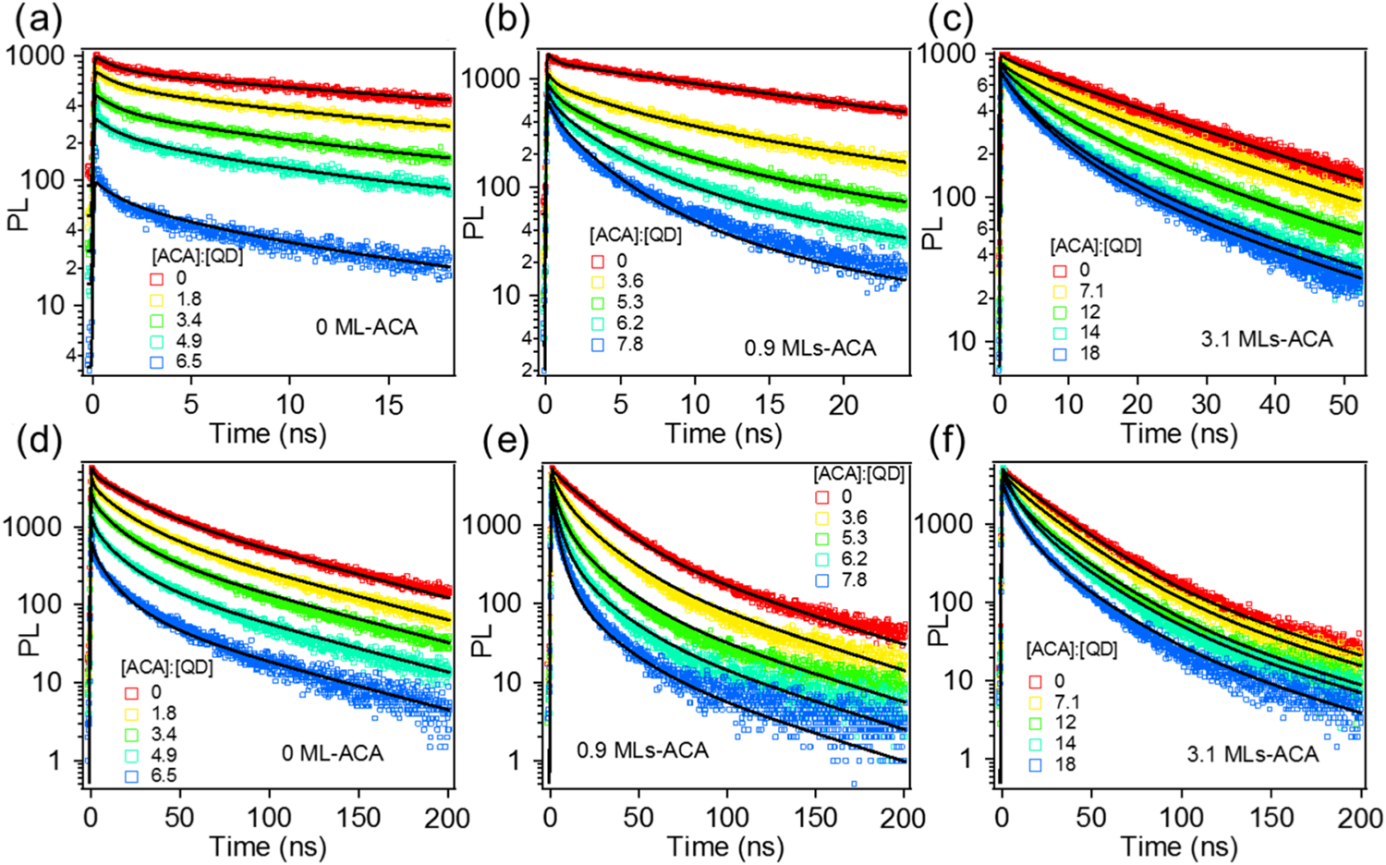
TRPL kinetics of (a) CdSe QD-ACA, (b) CdSe/CdS QD-ACA (0.9 monolayers of CdS), and (c) CdSe/CdS QD-ACA (3.1 monolayers of CdS) band edge emissions were observed with varying ACA concentrations (colored circles). (d), (e), and (f) are the corresponding TRPL kinetics traces collected in a longer detection time window (700 ns). The global fitting curves of the kinetics traces according to eqs S3 and S4 are shown as solid black lines. The excitation wavelength was set as 460 nm due to the limitation of the laser for TRPL.

In order to determine how the TET rate from the band edge state in core–shell QDs to ACA depends on the shell thickness, we turn to the TRPL measurement of band edge exciton decay kinetics. First, the band edge PL signal can be separated from trap state emissions to ensure that only band edge excitons are probed. Second, the TRPL kinetics of core–shell QD-ACA complexes are measured as a function of ACA concentrations, from which the TET rate from QD-ACA with one acceptor (referred to as intrinsic TET rate, *k*_i_) can be determined to allow meaningful comparison of TET rates in different samples. Two detection time windows (100 and 700 ns) were applied to resolve the band edge emission kinetics of QD-ACA in early and late time scales, respectively. The results are shown in Figure 3 and Figure S6 in **SI6**. With increasing ACA concentrations, QD-ACA complexes show faster band edge PL decay traces and smaller initial PL amplitudes. With thicker CdS shells, the TRPL traces show smaller initial PL amplitude loss and slower decays on the 1 to 100 ns time scale. The initial amplitude recorded by the PL decay reflects the exciton decay within the <0.1 ns time scale. Because the TA measurements above show negligible TET in these complexes within 0.1 ns, the initial amplitude loss in TRPL decay can be attributed to fast electron trapping induced by ACA adsorption. The slower TRPL decay component can be attributed to TET from the QD to the acceptors, consistent with the formation of the ACA triplet absorption shown by the TA measurement above. The TRPL kinetics detected in the two time windows are globally fitted assuming a Poisson distribution of ACA on QD surfaces to obtain the intrinsic TET rate.^60^ Details of the fitting are discussed in SI7. The fitting results are shown in Figure 3 and Figure S6, and the fitting parameters are summarized in Tables S1–S5 in SI7. All TRPL kinetics were fit well according to the model in SI7, except the fast decay at <2 ns in Figure 3a with the [ACA]:[QD] ratio of 6.5, which may be caused by extra electron trapping due to ACA adsorption.

### Coupling Strengths of TET, Electron Transfer, and Hole Transfer

TET from CdSe/CdS QDs to ACA is considered to proceed with the Dexter energy transfer mechanism.^14,43^ The dependence of TET rate (*k*_i_) on coupling strength can be described as
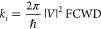
1where |*V*|^2^ and FCWD are the coupling strength for TET and the Franck–Condon overlap weighted density of states, respectively.^43^ In molecular donor–acceptor systems, the TET-coupling strength consists of two-electron exchange integral and one-electron integral term from the charge transfer virtual state:
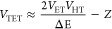
2where *V*_ET_, *V*_HT_, and *Z* are the electron transfer coupling strength, hole transfer coupling strength, and exchange integral, respectively, and Δ*E* is the energy difference between the charge transfer virtual state and the donor excited state.^48^ The first term is considered to be dominant in eq 2 for molecular donor–acceptor systems,^49^ and *Z* is approximately proportional to the product of *V*_ET_ and *V*_HT_ when the wave function is localized compared to the donor and acceptor distance.^47^ If the coupling strength of TET from CdSe/CdS QDs to the acceptor is proportional to product of *V*_ET_ and *V*_HT_ as in molecular donor–acceptor systems, then the coupling strength decay constant β_V_ along the donor–acceptor distance for TET is expected to be equal to the sum of βs for electron and hole transfers.^46^ To test the relationship between TET-coupling strength and electron/hole transfer coupling strength in the CdSe/CdS QD system, we also measured the intrinsic electron/hole transfer rates (*k*_i_) as a function of CdS shell thickness (with details in SI8 and SI9), from which the shell thickness dependence of the electron/hole transfer coupling strength can be extracted.^25^ MV^2+^ and PTZ were selected as electron and hole acceptors, respectively. *k*_i_ of electron/hole transfer from core–shell QDs to MV^2+^/PTZ were determined from the fitting of 1S_h_–1S_e_ XB kinetics in TA and band edge emission kinetics in TRPL with increasing loading of MV^2+^ and PTZ, respectively (Figures S8 and S11). The results are shown in Tables S6–S11 in the SI8 and SI9. We note that both the intrinsic electron transfer and hole transfer show multiexponential decay due to their heterogeneous nature.^68,69^ In order to better represent these nonsingle exponential decay processes, the rate constants corresponding to the half lifetime of these decay processes, *k*_1/2_, are calculated (see details in SI8) and used in the following discussion of the coupling strength. We also note that different rate constant analysis methods show negligible influences on the shell thickness dependence of the electronic coupling discussed below.

The measured ET rate changes exponentially from 2108 to 17 ns^–1^ as the shell thickness ranges from 0 to 1.3 nm (Tables S6–S10), with an exponential rate decay constant of β_*k*_ET__ = 0.32 Å^–1^ (Figure S12). In earlier experimental studies, Wachtveitl^70^ reported a β_*k*_ET__ value of 0.33 Å^–1^ and Smith^71^ reported β_*k*_ET__ = 0.13 Å^–1^ for ET between CdSe/CdS core–shell QDs and MV^2+^. The reported ET rate decay constant varies between 0.1 to 0.3 Å^–1^, depending on the experimental conditions (including the QD size and solvent), and the measured ET rate decay constant β_*k*_ET__ in our study falls within the reported range. Our ET couplings were extracted from the measured rates using an Auger-assisted model. Previous experimental studies of ET in QD-molecule assemblies show that the ET rate derived using this model agrees closely with measured charge transfer rates for ET in the Marcus-inverted regime:^64,72,73^
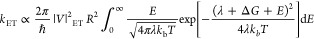
3

**Figure 4:**
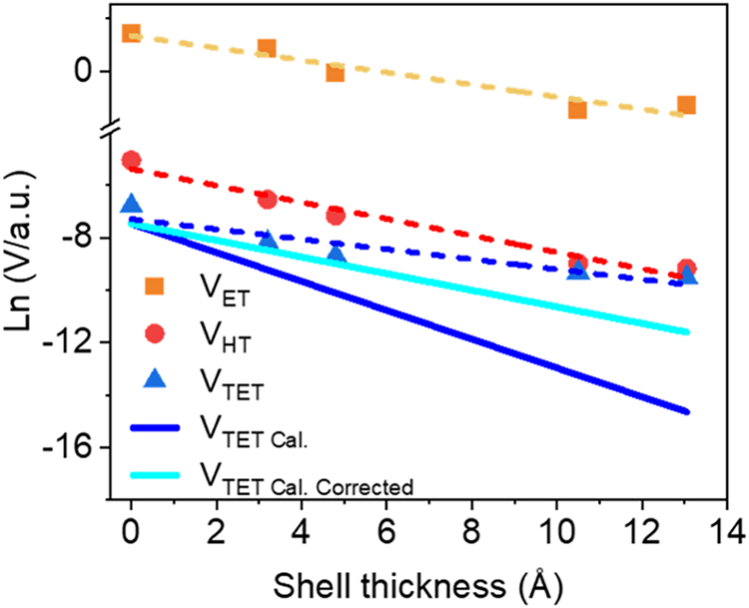
The ln value of the normalized coupling strength of ET (*V*_ET_, orange squares), HT (*V*_HT_, red dots), and TET (*V*_TET_, blue triangles) extracted from eqs 3, 4, and 5, respectively, using the measured rate constants. The dashed lines are linear fits to the ln value with slopes of β_V,ET_ = 0.18 Å^–1^, β_V,HT_ = 0.29 Å^–1^, and β_V,TET_ = 0.19 Å^–1^. The blue solid line represents the calculated V_TET_ (*V*_TET Cal._) assuming β_V,TET_ = β_V,ET_ + β_V,HT_. The cyan solid line represents the correction of *V*_TET Cal._ under the effect of shell thickness distribution broadening (*V*_TET Cal. Corrected_). Both *V*_TET Cal._ and *V*_TET Cal. Corrected_ are scaled for better comparison with *V*_TET_.

In eq 3, |*V*|_ET_^2^ is the squared electronic coupling between the QDs and MV^2+^, and *R* is the QD radius. In the Auger-assisted model, the ET is accompanied by hole excitation to higher valence band states due to the enhanced electron–hole coupling in the confined QD. As a result, in eq 3, the overall driving force is modified by the excited hole energy *E* in the product state, and the *R*^2^ term results from the QD radius dependence of the electron–hole coupling and the hole density of states in the valence band near band edge.^64^ The inner sphere reorganization energy for MV^2+^ is estimated to be 0.3 eV based on density functional theory (DFT) analysis (SI11); this contribution dominates the reorganization energy. The QDs are expected to have a small inner sphere reorganization energy (less than 10 meV).^74^ We assume that the total solvent and QD inner sphere reorganization energy is 0.1 eV^73,75^ and is independent of the shell thickness. The reaction free energy for ET is shell-thickness-dependent, as the CdSe/CdS has quasi-type II band alignment with a shallow potential barrier associated with the shell. As such, the electron in the conduction band 1S_e_ state delocalizes into the shell. The computed conduction band 1S_e_ orbital energies are −3.42, −3.57, −3.60, −3.68, and −3.70 eV, for 0, 0.32, 0.48, 1.05, and 1.3 nm of CdS shells, respectively (Table S13). The reduction potential energy for MV^2+^ is reported to be −4.4 V vs vacuum.^76^ The ET coupling strength, *V*_ET_, estimated from the measured ET rates using eq 3 for different shell thicknesses is shown in Figure 4. These couplings are fit to an exponential decay constant of β_V,ET_ = 0.18 Å^–1^. This decay constant is also computed using the analytical wave functions, assuming that the ET donor and acceptor are weakly coupled, so that the coupling is proportional to the surface density of the donor wave function, i.e., , where *A* is the surface area of the QD shell. The computed ET coupling distance decay constant is 0.21 Å^–1^, which is qualitatively consistent with the decay constant fit to the experimental data.

The experimentally determined HT rate from CdSe/CdS core/shell QDs to PTZ ranges from 20 to 1 ns^–1^ for QD shell thicknesses from 0 to 1.3 nm (Table S11). These rates are much slower than the corresponding ET rates. The computed valence band edge energies are −5.97, −5.91, −5.90, −5.90, and −5.90 eV for QDs with 0, 0.32, 0.48, 1.05, and 1.3 nm CdS shells, respectively (Table S13). The reduction potential energy of PTZ is reported to be −5.5 V vs vacuum.^69^ The inner sphere reorganization energy of PTZ is computed to be 0.087 eV (SI11), and we assume that the total solvent and QD inner sphere reorganization energy is 0.1 eV. The hole transfer is in the Marcus-inverted regime for all shell thicknesses. Fitting to the Auger-assisted model:^77^
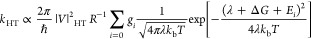
4where |*V*|_HT_^2^ is the squared coupling for hole transfer, and *g*_*i*_ is the degeneracy of the upper conduction band state with excess energy of *E*_i_ compared to the 1S_e_ state. The *R*^–1^ term represents the shell thickness dependence of the electron–hole coupling in the Auger-assisted model.^64^ The calculated energies of electrons at the corresponding 1S, 1P, 1D, and 2S levels for CdSe/CdS QDs are shown in Table S14 in **SI 10**. Using experimentally measured rates, the normalized HT couplings were extracted and fitted to the exponential decay with a decay constant of β_V,HT_ = 0.29 Å^–1^, as shown in Figure 4. Using our computed 1S_h_ hole state wave functions, we found a qualitatively consistent HT distance decay constant for the coupling of β_V,HT_ = 0.36 Å^–1^ by using the same procedure for calculating the ET coupling decay constant. The hole transfer coupling decay is faster than the electron transfer coupling decay because the hole transfer tunneling barrier (through the CdS shell) is larger than the barrier for electron transfer (0.6 eV vs 0.22 eV), and the hole effective mass is larger than the electron effective mass (0.8 *m*_0_ vs 0.21 *m*_0_). The derived donor–acceptor ET and HT couplings are all proportional to the donor wave function amplitudes on the QD surface, indicating that the donor and acceptor are weakly coupled.

The measured TET rate of ∼0.06 ns^–1^ is found to be largely independent of the CdS shell thickness (Figure S12). The TET rate can be described by a Marcus-like theory (*vide infra*)
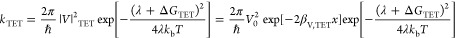
5Where β_V,TET_ is the TET-coupling decay constant, *V*_0_ is the coupling prefactor, and *x* is the QD shell thickness. Fitting measured rates to an exponential function of the shell thickness produces a TET rate decay constant of β_*k*_TET__ = 0.02 Å^–1^ (Figure S12). Since the TET rate is slow, the initially prepared states likely relax to the lowest exciton state prior to TET. The 1*S*_e_–1*S*_h_ QD excitation energy from both simulation and experiment depends strongly on the CdS shell thickness (Table S13). The TET reaction free energy (Δ*G*_TET_) therefore depends also on the shell thickness. The triplet excitation energy of ACA is 1.83 eV,^78^ leading to TET reaction free energies ranging from −0.59 to −0.35 eV for shell thicknesses varying from 0 to 1.3 nm. The solvent (nonpolar) reorganization energy for TET is very small,^79^ and the QD reorganization energy is also small,^74^ so we estimate the total reorganization energy as the inner sphere reorganization energy of ACA (0.22 eV).^79^ We also assume that this reorganization energy is independent of the shell thickness. The above reaction free energy and the reorganization energy data show that TET between the core–shell QDs and ACA is in the Marcus-inverted regime. However, as the shell thickness grows, the TET becomes less inverted. An inner sphere reorganization energy for ACA of 0.22 *eV* gives the coupling decay constant β_V,TET_ = 0.19 Å^–1^ by fitting the measured rates as a function of shell thickness. This fitted β_V,TET_ is much smaller than the theoretical value of about 0.5 Å^–1^, the sum of β_V,ET_ and β_V,HT_ (Figure 4). We note that β_V,TET_, β_V,ET_, and β_V,HT_ are measured by using different molecular acceptors with different binding configurations and specific distances to the QD surface. A detailed characterization of the surface molecular acceptors may exceed the scope of this work. However, they can be considered as independent of the QD shell thickness, given the similar ligand shell when increasing the CdS shell thickness. Therefore, although the absolute coupling strength may vary with different acceptors, the shell thickness dependence of the coupling, β_V_, is considered independent of the specific molecular acceptor and can be compared among ET, HT, and TET. We now discuss possible mechanisms that could account for the difference between computed and experimentally determined TET distance decays.

### Influence of the QD Size Distribution on the Reaction Free Energy and TET Couplings

**Figure 5:**
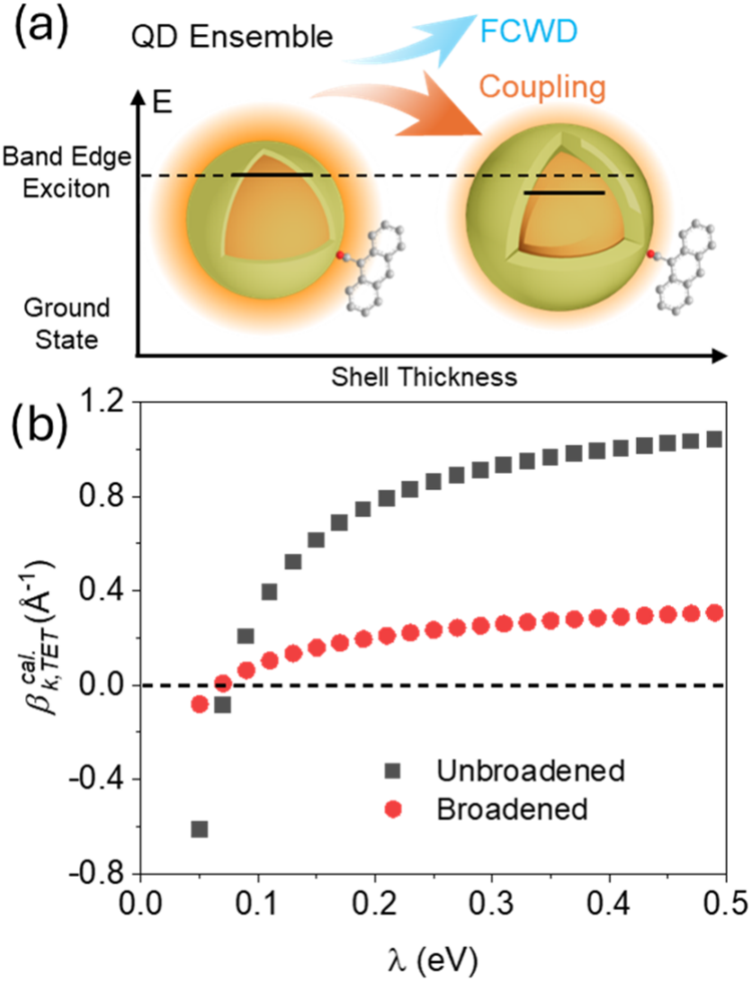
(a) Schematic of the shell thickness-dependent TET coupling and reaction free energy. The TET coupling (or QD surface density of exciton wave function, orange shadow) decreases exponentially with a thicker shell. The band edge exciton energy also decreases with a thicker shell, resulting in an exponential increase of the Franck–Condon overlap weighted density of states (FCWD) in the Marcus-inverted regime. In the shell thickness-dependent study, both trends are softened due to the heterogeneous size distribution of QD ensembles. (b) Simulated TET rate decay constant (β_k,TET_^cal.^) as the function of reorganization energy (λ) calculated by Marcus equation with (black squares) and without (red dots) the shell thickness distribution broadening effect.

In the weak coupling limit, the TET coupling has an approximately exponential dependence on the donor–acceptor distance, and smaller donor–acceptor distances associated with thinner shells will exponentially enhance the TET coupling. For an ensemble of QDs with varying shell thicknesses, a large standard deviation in the shell thickness distribution increases the number of QDs with thin shells (shorter donor–acceptor distances). These shorter donor–acceptor distances will increase the ensemble-averaged TET coupling (Figure 5a) and reduce the averaged TET coupling decay constant β_V,TET_. Since the QD exciton energy depends on the QD size, the average TET reaction free energy also depends on the QD size distribution. The TET rate, averaged over the shell thickness distribution (assuming a Gaussian distribution), includes the influence of the coupling and the reaction free energy on the rate:
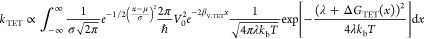
6

Here, μ and σ are the mean and standard deviation of the QD shell thickness, respectively, and Δ*G*_TET_ (*x*) is the shell thickness-dependent reaction free energy. Δ*G*_TET_ (*x*) is obtained by fitting the measured exciton energy spectrum of the QDs as a function of shell thickness (Figure S14). The reorganization energy is assumed to be independent of the shell thickness. We further assume that the QD core size distribution is the same for all CdSe/CdS core/shell QDs since the cores are all synthesized in a single batch. The mean shell thickness is calculated by subtracting the mean diameter of the bare CdSe core from that of core/shell QDs and dividing the result by 2. The standard deviation of QD shell thickness , where σ_QD_ and σ_core_ are the standard deviation of core/shell QD and bare CdSe core diameters, respectively. Thus, from the TEM measurement in Figures S1 and S2, the standard deviation of the shell thickness is 0.38, 0.38, 0.55, and 0.51 nm in core/shell QDs with 0.9, 1.4, 3.1, and 3.8 monolayers of CdS shell. Figure 5b shows the computed TET rate decay constants β_*k*_TET__ as a function of reorganization energy with and without considering the QD shell thickness. The QD exciton energy decreases as the shell thickness increases, producing an exponential increase in the Franck–Condon factor since TET occurs in the Marcus-inverted regime, as illustrated in Figure 5a. When the reorganization energy is smaller, the Franck–Condon factor grows more rapidly as a function of shell thickness. For very small reorganization energy values, the TET rate is expected to increase exponentially with shell thickness (i.e., a negative decay constant results). Comparing the averaged result to the TET rate decay constant computed without averaging over the shell thickness distribution, the QD size distribution reduces the computed TET rate decay constant by more than 70% for λ values larger than 0.1 eV. Using the reported 0.22 eV TET reorganization energy, the QD size distribution effect reduces the computed TET rate decay constant from 0.8 to 0.2 Å^–1^. However, the softened TET rate decay constant of 0.2 Å^–1^ is still much larger than the 0.02 Å^–1^ value derived from the measurements. Nonetheless, this analysis shows that uncertainties in the reorganization energy and the size-dependent reaction free energy produce large effects on the distance-dependent TET.

With a reorganization energy of 0.08 eV (Figure 5b), the shell thickness-broadened TET model produces a rate decay constant of 0.02 Å^–1^. Although a total reorganization energy of 0.08 eV is not realistic for TET between CdSe/CdS core–shell QDs and ACA species, this result shows that a reaction free energy change could also reduce the TET rate decay constant. The cited ACA reorganization energy of 0.22 eV is computed in vacuum.^79^ When adsorbed on the QD, the reorganization energy may change. The triplet excitation energy of ACA used in the Marcus equation was derived from measured spectra for free ACA species.^78^ On binding to QDs, the ACA excitation energy may change, leading to a shift in the TET reaction-free energy. As well, the QD size distribution may not be Gaussian and that could produce further changes in ensemble-averaged distance-dependent rates.

We also explored a multistate Marcus model that includes contributions to the observed kinetics that may arise from high-energy exciton states near the band edge; these high-energy states could be accessed by thermal fluctuations or vibronic broadening. Including higher energy exciton states would make the TET process more inverted, thus increasing the TET rate decay constant.

Shell thickness distribution-broadening effects influence ET, HT, and TET kinetics, and the effects arise from both the shell thickness-dependent donor–acceptor coupling and the reaction free energy, as illustrated in Figure 5a. First, the size distribution effects on shell thickness-dependent ET and HT couplings are expected to be weak. Considering the broadening effects, the averaged squared coupling  is
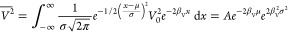
7where μ and σ are mean and standard deviation of the shell thickness distribution, *V*_0_^2^ and β_V_ values are the squared coupling prefactor and decay constant of the electronic coupling (ET, HT, and TET), respectively. Softening of the electronic coupling that arises from an increased shell thickness standard deviation is proportional to the squared coupling decay constant (β_*V*_^2^σ^2^). Since β_TET_ ≈ β_ET_ + β_HT_, the shell thickness distribution produces stronger softening effects for the TET coupling decay than for ET and HT. The computed ET, HT, and TET coupling decay constant for CdSe/CdS core–shell QDs are 0.21, 0.36, and 0.57 Å^–1^, respectively. The broadening effect reduces the decay constant of *V* for ET, HT, and TET to 0.18, 0.26, and 0.32 Å^–1^ (Figure 4). Thus, softening of the coupling decay arising from distributed shell thicknesses is more pronounced for the TET.

For a constant standard deviation of the shell thickness distribution for all QD sizes and assuming that the shell thickness distribution has no influence on the reaction free energy and the reorganization energy, the shell thickness-broadening effect produces uniform effects on the TET rates for all QD sizes. These effects will not change the intrinsic rate distance decay constant (except through the coupling prefactor). However, for core/shell QDs, the reaction free energy is shell thickness-dependent because of shell-induced changes of the QD wave function delocalization, leading to an exponential dependence of both the coupling and the Franck–Condon factors. These changes alter the rate’s distance decay constant. Including the broadening effect and using a reorganization energy of 0.22 eV, the TET Franck–Condon factor increases exponentially with shell thickness, with a growth constant of 0.14 Å^–1^. This value is smaller by about 0.05 Å^–1^ in comparison to the value found without considering the thickness-broadening effects. Overall, the TET-coupling decay constant dominates the softening of the TET rate decay constant in the CdSe/CdS-ACA system under study. Note that the strength of this broadening effect depends on the reorganization energy. For TET or charge transfer in a general core/shell QD system, the shell thickness distribution-broadening effect should be taken into account when computing both the effective electronic coupling and Franck–Condon factors. The extracted coupling strength from the experiments is the average of individual couplings. If the standard deviation of the tunneling medium thickness (molecule or semiconductor shell) is large, the average coupling is larger than that with the mean thickness. This is due to the exponential behavior of the coupling distance dependence. If the tunneling medium has a varying length or thickness distribution, the coupling decay constant will be influenced as well. The shell thickness distribution will also influence the reaction free energy, if the QD wave function has considerable delocalization in the shell, such as type I alignment with small band edge differences.

## Conclusions

In summary, we have tested whether the coupling strength of the core–shell QD-sensitized TET can be described by the model of the TET involving the charge transfer virtual state and whether the process can be considered as simultaneous electron and hole transfer. By varying the CdS shell thickness in CdSe/CdS QDs and extracting the shell thickness-dependent coupling strength of TET from QDs to molecular acceptor from the measured TET rates, we have demonstrated the exponential decrease of TET coupling strength with increasing shell thickness, and the exponential decay factor or damping coefficient β is 0.19 Å^–1^. We found that the TET rates for the CdSe/CdS core–shell structures are described by Marcus-like rates, including the shell thickness distribution and its impact on the Marcus rate parameters (free energy and coupling). The Franck–Condon factor is sensitive to the shell thickness-dependent reaction free energy and the reorganization energy, while the QD size distribution also modifies the TET rate through shell thickness-dependent TET coupling. By considering the QD size distribution-induced softening effect on the size dependence of the coupling strengths of TET, electron transfer, and hole transfer, we confirmed the TET mechanism of simultaneous electron and hole transfer in the QD-acceptor system. A similar impact of heterogeneity is anticipated in other energy or charge transfer systems utilizing inorganic nanoparticles with less controlled particle properties. This work suggests that it is important to consider the particle heterogeneity in the mechanism studies of such systems.

## Supporting Information Available

Figures S1–S14, Tables S1–S16, sample synthesis and experimental setups, details for EMA calculation and calculation of reorganization energy, details in fitting of TRPL kinetics of CdSe/CdS QDs-ACA, and determination of rates of electron transfer and hole transfer from CdSe/CdS QDs to the corresponding acceptors (PDF)
