# Supplementary material for: Shell Thickness and Heterogeneity Dependence of Triplet Energy Transfer between Core–Shell Quantum Dots and Adsorbed Molecules
Source: J Am Chem Soc. 2025 Apr 30;147(19):16282–92. doi: 10.1021/jacs.5c01838 (PMC12082699; doi:10.1021/jacs.5c01838)
Supplement: Supplementary file 1 — ja5c01838_si_001.pdf [file ja5c01838_si_001.pdf]

Supporting Information of

Shell Thickness and Heterogeneity Dependence of Triplet Energy Transfer between Core-shell Quantum Dots and Adsorbed Molecules

Tao Jin[1◊], Zhendian Zhang[2◊], Sheng He[1◊], Alexey L. Kaledin[3], Zihao Xu[1], Yawei Liu[1], Peng Zhang[2], David N. Beratan[2,4,5*], Tianquan Lian[1*]

[1]Department of Chemistry, Emory University, 1515 Dickey Dr, Atlanta, GA, 30322, USA

[2]Department of Chemistry, Duke University, Durham, NC 27708

[3]Cherry L. Emerson Center for Scientific Computation, Emory University, 1515 Dickey Dr, Atlanta, GA, 30322, USA

[4]Department of Physics, Duke University, Durham, NC 27708

[5]Department of Biochemistry, Duke University, Durham, NC 27710

Corresponding Author:

Tianquan Lian, email: tlian@emory.edu

David N. Beratan, email: david.beratan@duke.edu

[◊]T. J., Z. Z., and S. H. contributed equally.

Content List

SI1. Sample Preparation and Experimental Setups....3

SI2. Transmission Electron Microscopy (TEM) of CdSe/CdS Core-shell Quantum Dots (QDs) 5

SI3. Transient Absorption (TA) Spectra of CdSe/CdS QDs....7

SI4. UV-vis Spectra of CdSe/CdS QDs and CdSe/CdS QDs-9-Anthracenecarboxylic Acid (ACA) Complexes for TA Experiment....8

SI5. TA Spectra and Kinetics of CdSe/CdS QD-ACA (0, 1.4, 3.1 and 3.8 monolayers of CdS shell)....8

SI6. Time-resolved Photoluminescence (TRPL) Kinetics of Band Edge Emission of CdSe/CdS (1.4 Monolayers) QD-ACA and CdSe/CdS (3.8 Monolayers) QD-ACA....10

SI7. Fittings of TRPL Kinetics of CdSe/CdS QDs-ACA....10

SI8. Determination of Rates of Electron Transfer from CdSe/CdS QDs to Methyl Viologen (MV^{2+})....13

SI9. Determination of Rates of Hole Transfer from CdSe/CdS QDs to Phenothiazine (PTZ) 18

SI10. Core/shell QDs wave functions and excitation energies....21

SI11. Calculation of Reorganization Energies of the Acceptors for Electron/hole Transfer 23

SI1. Sample Preparation and Experimental Setups

Synthesis of CdSe/CdS QDs and Preparation of CdSe/CdS QD-ACA, CdSe/CdS QD-MV^{2+} and CdSe/CdS QD-PTZ Complexes

Cadmium oxide (CdO, 99.5%), selenium powder (Se, 100 mesh, 99.99%), Sulfur powder (S, 99.98%), 9-anthracenecarboxylic acid (ACA, 99%), tri-n-octylphosphine oxide (TOPO, 99%), tri-n-octylphosphine (TOP, 97%), oleic acid (OA, 90%), 1-octadecene (ODE, 90%), 1-octanethiol (OctSH, 99%), methyl viologen dichloride hydrate (MV^{2+}, 98%) and phenothiazine (PTZ, 98%) and all other solvents were purchased from Sigma-Aldrich. N-octadecylphosphonic acid (ODPA, 99%) was purchased from PCI Synthesis. These chemicals were used without any further purification.

The synthesis of CdSe QDs was performed following the procedure reported in previous literature.[1, 2] 60 mg CdO, 3 g TOPO and 280 mg ODPA were mixed in a 25 mL three-neck bottle. After being vacuumed at 60 ℃ for 20 minutes, the mixture was heated to 360 ℃ under Argon flow. 1 mL TOP was injected into the system after the mixture became colorless solution. The temperature was raised back to 360 ℃, and 0.5 mL Se TOP solution with 60 mg Se was quickly injected into the Cd solution. Once the color of the solution turned to orange, the reaction was quenched by intense cool air flow. After the temperature was decreased to 100 ℃, methyl acetate was added to precipitate the CdSe QDs. The QDs were further washed with hexane and methyl acetate for once and were finally dissolved in hexane with the concentration of 9.3×10^{-5} M. The CdSe QD solution was stored in a glove box with argon atmosphere for further use.

CdS shell was grown on CdSe QDs by the method reported in previous literature.[1, 2] 0.2 M cadmium oleate ($Cd(OA)_2$) in ODE was prepared as Cd precursor solution. 257 mg CdO, 6.31 mL OA and 3.66 mL ODE were added to a 25-mL three-neck bottle. The mixture was placed in vacuum at 60 ℃ for 20 minutes and then in Argon flow for 10 minutes, after which the temperature was raised to 230 ℃. After the solution turned colorless, the temperature was decreased to and maintained at 110 ℃. 0.2 M OctSH in ODE was prepared as S precursor solution by dissolving 0.347 mL OctSH in 9.653 mL ODE through ultrasonication. For the growth of CdS shell, 100 nanomoles CdSe QDs in solution were added to a 25-mL three-neck bottle with 5 mL ODE. Hexane and oxygen in the mixture were removed by vacuum, after which the temperature of the solution was raised to 240 ℃. Desired amount of Cd and S precursor solutions in two separate syringes were then slowly injected into the CdSe QDs solution at a rate of 3 mL per hour by two syringe pumps. The amount of Cd and S precursor solutions used to achieve growth of certain shell thickness was calculated by estimated CdSe QD core size and the CdS shell lattice constant. After injection of Cd and S solutions, the temperature was further increased to and maintained at 310 ℃ for 10 minutes. Then the temperature of the solution was decreased to 100 ℃ with cool air flow, and excess methyl acetate was added to precipitate the CdSe/CdS QDs. The core-shell QDs were further washed with hexane and methyl acetate and finally dispersed in 10 mL hexane or toluene with concentration of ~ 1×10^{-5} M. The core-shell QDs were stored in the glove box for further use.

The CdSe/CdS QD-ACA, CdSe/CdS QD-MV^{2+} and CdSe/CdS QD-PTZ complexes for TA experiment were prepared by adding 1 mg ACA/MV^{2+}/PTZ powder into 1 mL CdSe/CdS QDs so that the amount of acceptor was much larger than that of QDs. The mixture was ultrasonicated for 2 hours, and excess undissolved powders were filtered out. The solvents in the solutions were then removed by air flow, and the complexes were re-dispersed in hexane in the glove box for experiments. For preparation of CdSe/CdS QD-ACA, CdSe/CdS QD-MV^{2+} and CdSe/CdS QD-PTZ complexes for determination of

energy/electron/hole transfer rates with varying acceptor concentrations, varying amount of ACA/MV^{2+}/PTZ in toluene solutions (1×10^{-3} M) was added to 1 mL CdSe/CdS QDs toluene solutions. The concentration ratios of acceptors to QDs in the solution were shown in the main text and **SI 8** and **SI 9**. Specifically, the concentration ratios of ACA/PTZ to QDs were determined from UV-vis spectra of the solution, and the concentration ratios of MV^{2+} to QDs were determined from the calculation of amount of MV^{2+} added to the QDs solution and amount of QDs in solution, considering that the UV-vis spectra of MV^{2+} cannot be well resolved in the spectra window. The solutions were ultrasonicated for 2 hours, and the solvents were removed by air flow. Finally, the complexes along with free acceptors were re-dispersed in toluene in the glove box for TA/TRPL experiments.

Transient Absorption Spectroscopy Setups

The femtosecond transient absorption (TA) setup was based on a regenerative amplified Ti: Sapphire femtosecond laser system (Coherent Legend), which generates 800 nm fundamental pulse with pulse duration of 150 fs, repetition rate of 1 kHz and pulse energy of 2 mJ/pulse. The 800 nm pulse was directed through a 90:10 beam splitter. The 10% was focused onto a 2 mm thick sapphire crystal or a 3 mm thick CaF_2 crystal to generate the white light continuum as femtosecond TA probe. The spectra windows of the probe were 380-650 nm for CaF_2 crystal and 440-900 nm for sapphire crystal. The probe pulse was directed through a 70:30 beam splitter to generate the signal and reference probe. 1 W of 800 nm fundamental pulse was directed to an optical parametric amplifier (OPA). The signal output from the OPA was aligned with the remaining 800 nm fundamental at a BBO crystal to generate the 520 nm pump pulse through sum frequency generation. The remaining signal pulse and fundamental pulse after the BBO crystal were filtered out with a band pass filter. The pump went through a 500 Hz chopper as a modulation to provide the sample signals with and without the pump pulse excitation. A delay stage was applied for the pump pulse to adjust the time delay between the pump and the probe pulse. The pump and probe pulses were focused onto the sample with diameters of 400 μm and 100 μm, respectively. The power density of the pump pulse on the sample was 160 $\mu J/cm^2$/pulse. The probe beams were finally focused into optical fibers coupled with a visible spectrometer and a CMOS camera with 1024 elements. The data was collected with the Helios system from Ultrafast Systems, Inc. Instrument response function was fit to be a Gaussian function from the solvent response. The time window of the femtosecond TA is up to 1.6 ns with 200 fs time resolution.

For nanosecond TA experiments, the pump pulse from the femtosecond TA setups was directed and focused onto the sample without going through the 500 Hz chopper. The probe pulse was generated by a white light continuum laser (STM-2-UV, Leukos) with pulse duration of 0.5 ns and repetition rate of 2kHz. The data was collected by the EOS system from Ultrafast Systems, Inc.

The samples for the experiments were added to 1 mm thick quartz cuvettes (Starna) and were constantly stirred during TA experiment.

Steady State Photoluminescence Setups

Steady state photoluminescence experiments were performed on a Fluoromax fluorometer (Horiba Scientific). The excitation wavelength was set to be 400 nm for CdSe core QDs and 510 nm for CdSe/CdS core-shell QDs. The photoluminescence quantum yields of CdSe and CdSe/CdS QDs were determined with the method reported in previous literature with Coumarin 153 and Rhodamine 6G as standards, respectively.

1 cm cuvettes were applied for measurements.

Time-resolved Photoluminescence (TRPL) Setups

TRPL experiments were based on a mode-locked Ti:Sapphire laser (Tsunami oscillator, Spectra Physics). The wavelength of the output pulse was tuned to be 920 nm. The time duration of the pulse was 100 fs and the repetition rate was 80 MHz. The repetition rate was reduced by a pulse picker (Conoptics) to 20 MHz, 10 MHz and 1.43 MHz for the detection time windows of 50 ns, 100 ns and 700 ns, respectively. The pulse going through the pulse picker was directed to a BBO crystal to generate the 460 nm excitation pulse through second harmonic generation. The remaining 920 nm pulse was filtered out by band pass filters. The 460 nm pulse was directed to the sample, and the emission was collected into a monochromator (Acton Series, Princeton Instruments), by which the band edge exciton emission of the QDs was selected out. The emission going through the monochromator was collected and amplified by a microchannel-plate-photomultiplier tube (Hamamatsu R3809U-51) and analyzed by a time-correlated single photon counting (TCSPC) board (Becker & Hickel SPC 600). The instrument response function was measured to be a Gaussian function with FWHMs of 62 ps, 110 ps and 626 ps for 50 ns, 100 ns and 700 ns detection time windows, respectively. The absorption at 460 nm, the data collection time and the excitation pulse intensity were controlled to be the same for QDs with and without the acceptors.

SI2. Transmission Electron Microscopy (TEM) of CdSe/CdS Core-shell Quantum Dots (QDs)

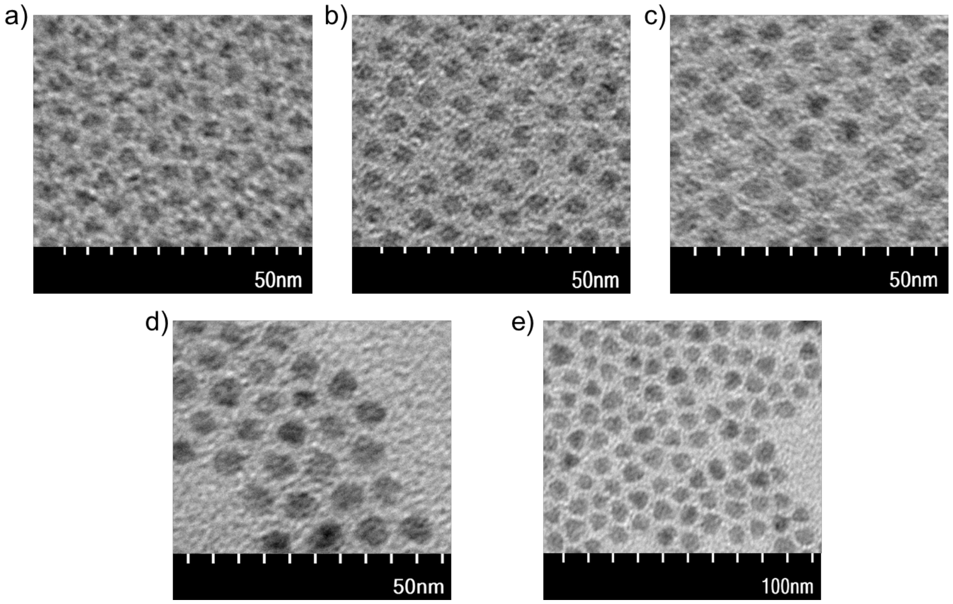

Figure S1: Parts of the TEM images of CdSe/CdS core-shell QDs with a). 0, b). 0.9, c). 1,4, d). 3.1 and e). 3.8 monolayers of CdS shell.

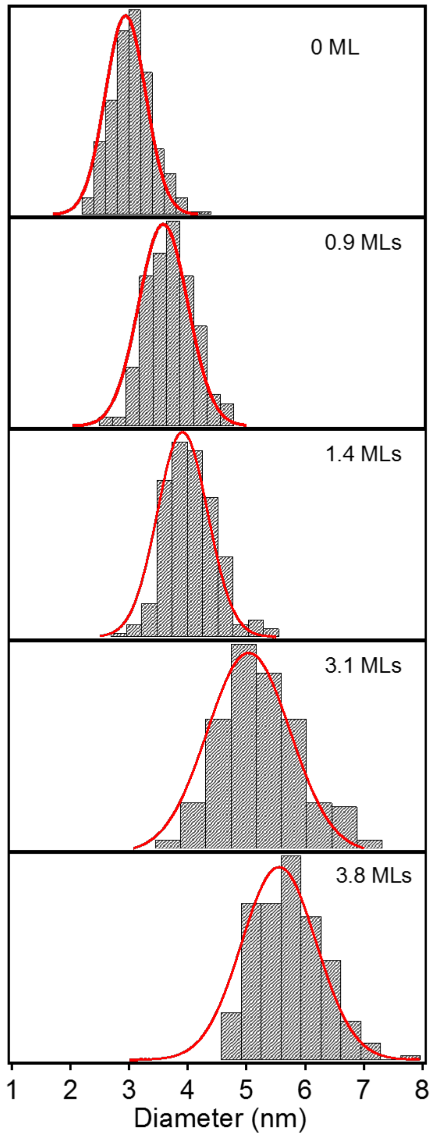

Figure S2: Size distributions of the synthesized CdSe QDs and CdSe/CdS core-shell QDs extracted from the TEM images. The red solid lines are the fits to the diameter histograms by Gaussian distribution. The average diameters (standard deviation) of the QDs to be 2.94 (0.49) nm, 3.58 (0.59) nm, 3.90 (0.60) nm, 5.04 (1.00) nm and 5.55 (0.90) nm, corresponding to 0, 0.9, 1.4, 3.1 and 3.8 monolayers of CdS shell, respectively. (1 monolayer = 0.35 nm).[3]

SI3. Transient Absorption (TA) Spectra of CdSe/CdS QDs

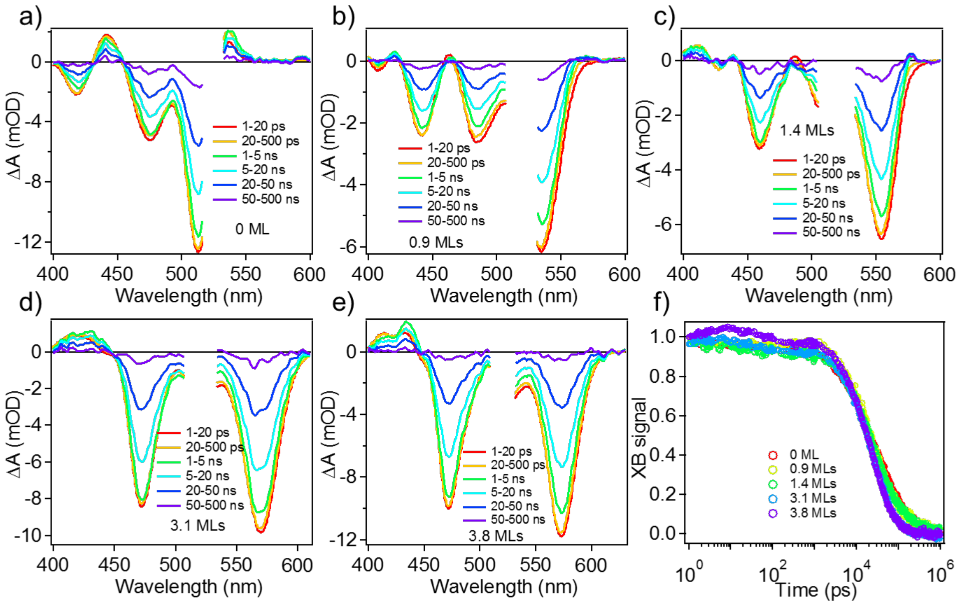

Figure S3: TA spectra of the synthesized CdSe/CdS QDs with a). 0, b). 0.9, c). 1.4, d). 3.1 and e). 3.8 monolayers of CdS shell in the delay time range of 1 ps – 1 µs. The wavelength of the pump pulse was 520 nm. f). Exciton bleach kinetics of the $1S_h$-$1S_e$ core transition of the QDs.

Figure S3 shows the TA spectra of the synthesized CdSe core QDs and CdSe/CdS core-shell QDs excited by the 520 nm pump pulse. As shown in the UV-vis spectra of the QDs in **Figure 1**, 520 nm pump pulse excited the $1S_h$-$1S_e$ transition in CdSe/CdS QDs with 0, 0.9, and 1.4 monolayers of CdS shell and the $2S_h$-$1S_e$ transition in CdSe/CdS QDs with 3.1 and 3.8 monolayers of CdS shell.[4] For the latter two batches of QDs, the hole quickly relaxed to $1S_h$ level within 10 ps, which results in the growth of the exciton bleach (XB) signal of $1S_h$-$1S_e$ transition in **Figure S3f**.[4] As shown in **Figure S3a** to **Figure S3e**, TA spectra of the QDs contain spectra features of $1S_h$-$1S_e$ transition XB at 511 nm, 535 nm, 554 nm, 570 nm, and 573 nm for CdSe/CdS QDs with 0, 0.9, 1.4, 3.1, and 3.8 monolayers of CdS, respectively and the increasing amplitudes of the T band bleach at ~475 nm. As shown in **Figure S3f**, due to the decrease of the trap states by shell growth, XB decay consists more of the band edge exciton recombination, which is typically faster than recombination of band edge electron with trapped hole in un-passivated CdSe QDs.[5] Therefore, with increasing shell thickness, XB kinetics show faster decay.

SI4. UV-vis Spectra of CdSe/CdS QDs and CdSe/CdS QDs-9-Anthracenecarboxylic Acid (ACA) Complexes for TA Experiment

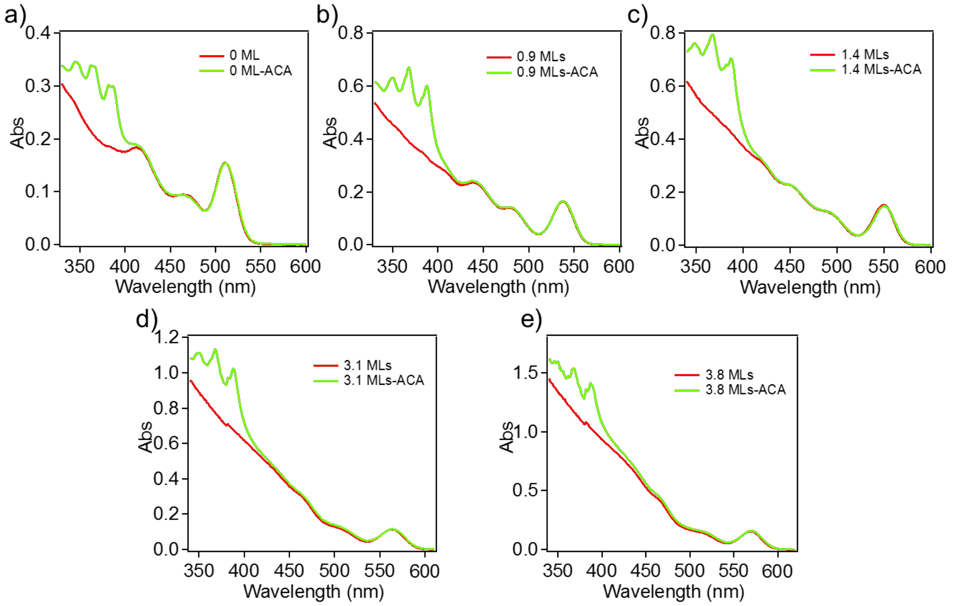

Figure S4: UV-vis spectra of CdSe/CdS QDs and CdSe/CdS QD-ACA complexes with a). 0, b). 0.9, c). 1.4, d). 3.1 and e). 3.8 monolayers of CdS shell for TA experiments. The spectra of CdSe/CdS QD-ACA contain absorption of both free ACA in solution and ACA attached to QD surfaces. The concentration ratios of total ACA to QD are 1.7, 5.2, 8.1, 14.4 and 13.1 for CdSe/CdS QDs with 0, 0.9, 1.4, 3.1 and 3.8 monolayers of CdS shell, respectively.

SI5. TA Spectra and Kinetics of CdSe/CdS QD-ACA (0, 1.4, 3.1 and 3.8 monolayers of CdS shell)

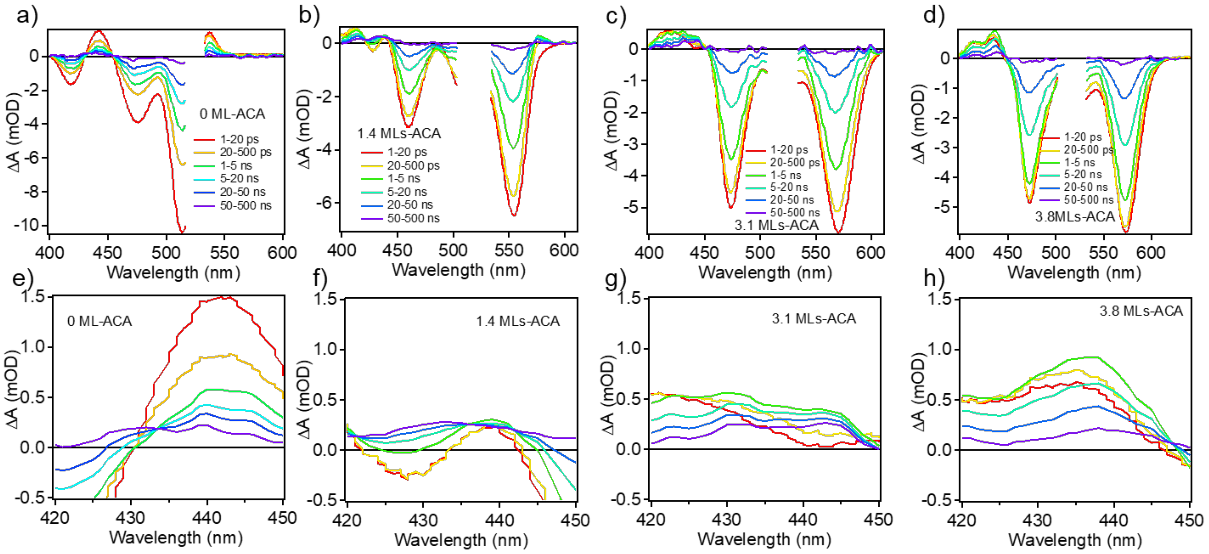

Figure S5-1: TA spectra of CdSe/CdS QD-ACA complexes with a). 0; b). 1.4; c). 3.1 and d). 3.8

monolayers of CdS shell. The delay time range is from 1 ps – 1 µs. The wavelength of the pump pulse was 520 nm. e)-f) shows the zoom-in plot of a)-d) respectively to resolve the ^{3}ACA* T_1->T_n TA signal in range of 400 nm – 450 nm.[6] The ^{3}ACA* signal suggests the triplet energy transfer from CdSe/CdS QDs to ACA.

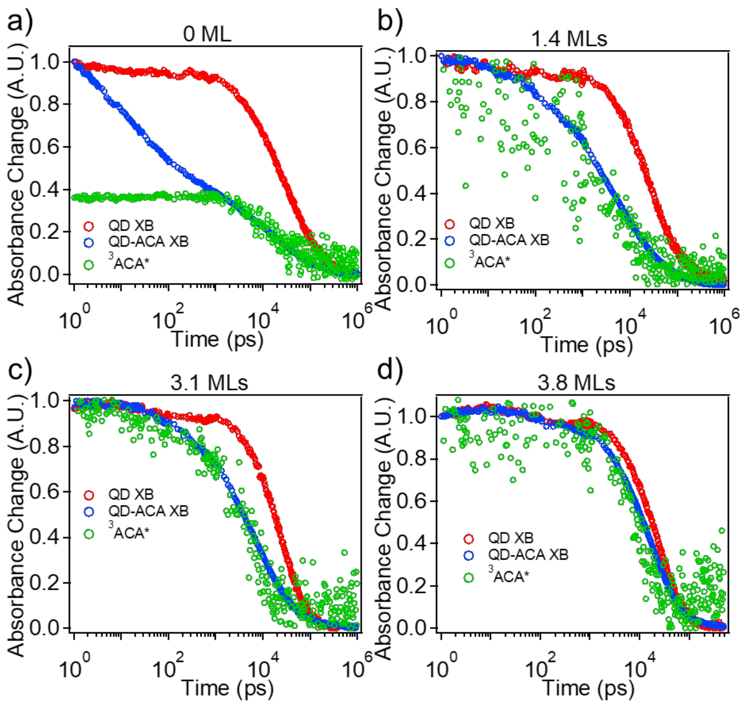

Figure S5-2: Normalized XB kinetics at the core $1S_h$-$1S_e$ transition for QDs (red) and QD-ACA complex (blue) along with ^{3}ACA* signal growth kinetics (green) in the time range of 1 ps – 1 µs. a), b), c) and d) correspond to CdSe/CdS QDs with 0, 1.4, 3.1 and 3.8 monolayers of CdS shell, respectively. The ^{3}ACA* growth kinetics were inverted and normalized to 1 in panel b)-d) for better comparison with QD-ACA XB decay. Due to extra XB decay within 1 ns through electron trapping for CdSe QD-ACA, the ^{3}ACA* growth kinetics was inverted and normalized to the XB amplitude after 1ns in panel a).

SI6. Time-resolved Photoluminescence (TRPL) Kinetics of Band Edge Emission of CdSe/CdS (1.4 Monolayers) QD-ACA and CdSe/CdS (3.8 Monolayers) QD-ACA

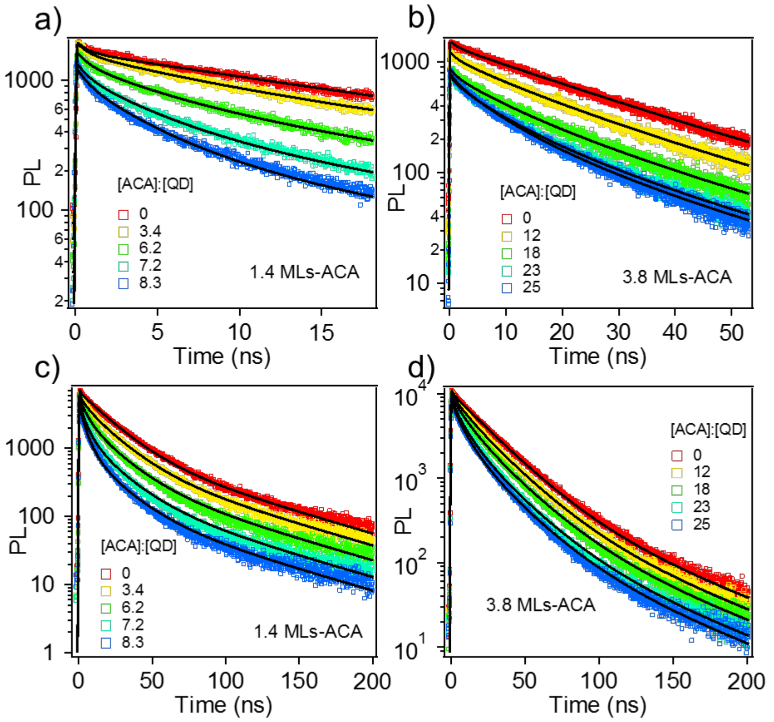

Figure S6: TRPL kinetics of a). CdSe/CdS QD-ACA (1.4 monolayers of CdS) and b). CdSe/CdS QD-ACA (3.8 monolayers of CdS) band edge emission with varying ACA concentrations (colored circles). c) and d) are the corresponding TRPL kinetics traces collected in longer detection time window (700 ns). The global fitting curves of the kinetics traces according to Eq. S3 and Eq. S4 are shown as solid black lines.

SI7. Fittings of TRPL Kinetics of CdSe/CdS QDs-ACA

In TRPL experiments, the wavelength of the excitation pulse was set as 460 nm due to limitation of the laser for TRPL. The excitation pulse would inevitably excite the T band transition in CdSe/CdS QDs. According to our previous research, the generated hole after excitation would either be trapped to surface or relax to $1S_h$ level localized in the core in much shorter time compared to TET process.[3] Here we only probed the band edge emission kinetics, thus the TRPL kinetics traces mainly show TET from band edge excitons. The instrument response functions (IRF) of the TRPL were characterized by Gaussian functions with FWHM of 110 ps and 626 ps for 100 ns and 700 ns time windows, respectively.

In order to fit these TRPL traces to extract the k_i, we assume the Poisson distribution of adsorbed ACA on QD surface:[7]

$$P(n,m) = \frac{m^n e^{-m}}{n!} \quad \text{(S1)}$$

where m and n are the average and specific numbers of adsorbed ACA on QDs. The apparent rate of TET to ACA in a QD-ACA complex with n adsorbed ACA is:[7]

$$k_{app}(n) = nk_i \quad \text{(S2)}$$

Accordingly, the TRPL traces of QD-ACA with 700 ns detection time window can be derived as:

$$N(t,m) = bN(0)(\sum_n e^{-nk_i t} P(n,m))S(t) \quad \text{(S3)}$$

where N(0) is the initial PL amplitude at time zero, reflecting the initial concentration of excited QDs with a band edge exciton, S(t) is the band edge exciton decay kinetics trace of QDs without ACA, which is described by a three-exponential decay function, and (1-b) is the initial PL amplitude loss due to fast electron trapping that cannot be resolved by our TRPL measurement. In the TRPL traces of QD-ACA with 100 ns time window, the observed signal includes the QD-ACA emission introduced by previous excitation pulses because of the shorter pulse repetition time than the QD exciton lifetime.[7] Therefore, the kinetics traces can be given by:[7]

$$N(t,m) = C\sum_j (bN(0)(\sum_n e^{-nk_i(t+jT_R)} P(n,m))S(t+jT_R)) \quad \text{(S4)}$$

where T_R is the pulse repetition time (100 ns), and C is the constant to account for the difference in excitation pulse energies and data acquisition times of TRPL experiments of 100 ns and 700 ns detection time windows. Eq. S3 and Eq. S4, after convolution with the Gaussian IRF, are used to globally fit the TRPL kinetics traces in **Figure 3** and **Figure S6**. In the global fitting, the summations of n were performed until n = 20, considering that the average numbers of adsorbed ACA on QD surface in TRPL experiments are expected to be smaller than 20, and the probability of QD with more than 20 adsorbed ACA, which follows Poisson distribution (Eq. S1), is negligible.[8] The summations of j in Eq. S4 were performed until j = 10 because the lifetime of the QD TRPL signal is smaller than 1000 ns, and the contribution of the excitation pulse arriving 1000 ns before the time of signal detection to the QD TRPL signal of 100 ns detection time window is negligible. TRPL kinetics traces of QDs without ACA can be fit with three-exponential function convoluted with the Gaussian IRF, and the parameters of the fitting were fixed for fitting of the kinetics traces of QD-ACA. The rates of TET from QD to one ACA molecule (k_i) were controlled to the same for QD-ACA with varying ACA concentrations, and m and b in Eq. S3 and Eq. S4 were controlled to the same for kinetics traces in 100 ns and 700 ns detection time windows of QD-ACA with specific ACA concentration. The fitting result is shown in **Table S1-Table S5**.

[ACA]:[QD]	0	1.8	3.4	4.9	6.5
a_1	$(1.80 \pm 0.02) \times 10^3$				
τ_1 (ns)	1.19 ± 0.02				
a_2	$(2.99 \pm 0.02) \times 10^3$				
τ_2 (ns)	20.1 ± 0.2				
a_3	$(1.91 \pm 0.02) \times 10^3$				
τ_3 (ns)	72.9 ± 0.5				
m	0	0.328 ± 0.003	0.548 ± 0.004	0.732 ± 0.009	1.13 ± 0.02
k_i ($k_{1/2}$) (ns^{-1})	0.0677 ± 0.0010 (0.0977 ± 0.0014)				
b	1	0.721 ± 0.001	0.461 ± 0.001	0.232 ± 0.001	0.112 ± 0.001
C	0.136 ± 0.001				

Table S1: Parameters of global fitting of TRPL kinetics traces of CdSe QD-ACA in **Figure 3a** and **Figure 3d**. a_i and τ_i (i = 1, 2, 3) are the parameters in three-exponential function to fit the kinetics traces of QD without ACA.

[ACA]:[QD]	0	3.6	5.3	6.2	7.8
a_1	$(1.37 \pm 0.03) \times 10^3$				
τ_1 (ns)	0.457 ± 0.013				
a_2	$(4.34 \pm 0.02) \times 10^3$				
τ_2 (ns)	18.8 ± 0.1				
a_3	754 ± 22				
τ_3 (ns)	62.3 ± 1.0				
m	0	0.711 ± 0.003	1.37 ± 0.01	1.98 ± 0.01	2.76 ± 0.02
k_i ($k_{1/2}$) (ns^{-1})	0.119 ± 0.001 (0.172 ± 0.001)				
b	1	0.943 ± 0.002	0.724 ± 0.002	0.594 ± 0.002	0.508 ± 0.002
C	0.268 ± 0.001				

Table S2: Parameters of global fitting of TRPL kinetics traces of CdSe/CdS QD (0.9 monolayers of CdS)-ACA in **Figure 3b** and **Figure 3e**.

[ACA]:[QD]	0	3.4	6.2	7.2	8.3
a_1	$(1.49 \pm 0.03) \times 10^3$				
τ_1 (ns)	0.654 ± 0.021				
a_2	$(5.77 \pm 0.02) \times 10^3$				
τ_2 (ns)	19.0 ± 0.1				
a_3	$(1.00 \pm 0.03) \times 10^3$				
τ_3 (ns)	69.4 ± 1.1				
m	0	0.275 ± 0.003	0.715 ± 0.004	1.13 ± 0.01	1.52 ± 0.01
k_i ($k_{1/2}$) (ns^{-1})	0.117 ± 0.001 (0.169 ± 0.001)				
b	1	0.880 ± 0.002	0.828 ± 0.002	0.716 ± 0.002	0.670 ± 0.002
C	0.233 ± 0.001				

Table S3: Parameters of global fitting of TRPL kinetics traces of CdSe/CdS QD (1.4 monolayers of CdS)-ACA in **Figure S6a** and **Figure S6c**.

[ACA]:[QD]	0	7.1	12	14	18
a_1	387 ± 14				
τ_1 (ns)	1.56 ± 0.09				
a_2	$(4.44 \pm 0.02) \times 10^3$				
τ_2 (ns)	23.8 ± 0.1				
a_3	320 ± 28				
τ_3 (ns)	74.9 ± 3.3				
m	0	0.231 ± 0.003	0.714 ± 0.004	1.17 ± 0.01	1.52 ± 0.01
k_i ($k_{1/2}$) (ns^{-1})	0.0825 ± 0.0007 (0.119 ± 0.001)				
b	1	0.933 ± 0.002	0.860 ± 0.002	0.790 ± 0.003	0.703 ± 0.002
C	0.184 ± 0.001				

Table S4: Parameters of global fitting of TRPL kinetics traces of CdSe/CdS QD (3.1 monolayers of CdS)-ACA in **Figure 3c** and **Figure 3f**.

[ACA]:[QD]	0	12	18	23	25

a_1	$(1.23 \pm 0.02) \times 10^3$				
τ_1 (ns)	1.34 ± 0.03				
a_2	$(9.61 \pm 0.03) \times 10^3$				
τ_2 (ns)	23.4 ± 0.1				
a_3	780 ± 36				
τ_3 (ns)	65.6 ± 1.4				
m	0	0.270 ± 0.002	0.519 ± 0.002	0.832 ± 0.003	1.02 ± 0.01
k_i ($k_{1/2}$) (ns^{-1})	0.0622 ± 0.0004 (0.0897 ± 0.0006)				
b	1	0.954 ± 0.001	0.912 ± 0.001	0.819 ± 0.001	0.789 ± 0.001
C	0.130 ± 0.001				

Table S5: Parameters of global fitting of TRPL kinetics traces of CdSe/CdS QD (3.8 monolayers of CdS)-ACA in **Figure S6b** and **Figure S6d**.

SI8. Determination of Rates of Electron Transfer from CdSe/CdS QDs to Methyl Viologen (MV^{2+})

MV^{2+} was applied as the electron transfer acceptor for determination of the shell thickness dependent electron transfer rate. As shown in **Figure S7**, electron transfer from CdSe/CdS QDs to MV^{2+} can be well resolved in TA spectra. For CdSe-MV^{2+}, charge separated state feature consisting of MV^+ radical absorption signal from 550 nm to 700 nm and derivative-like feature due to Stark effect is almost instantaneously formed after excitation of the QDs, suggesting ultrafast electron transfer from QDs to MV^{2+}.[3] The simultaneous decay of the MV^+ radical signal and the derivative-like feature at the XB position indicates charge recombination between the hole in the QD and the electron in MV^+. The formation of charge separated state signal is slower with increasing shell thickness, and the relative signal amplitude of MV^{2+} radical is smaller with the growth of CdS shell, which indicates the slower and less efficient electron transfer from the core-shell QDs.

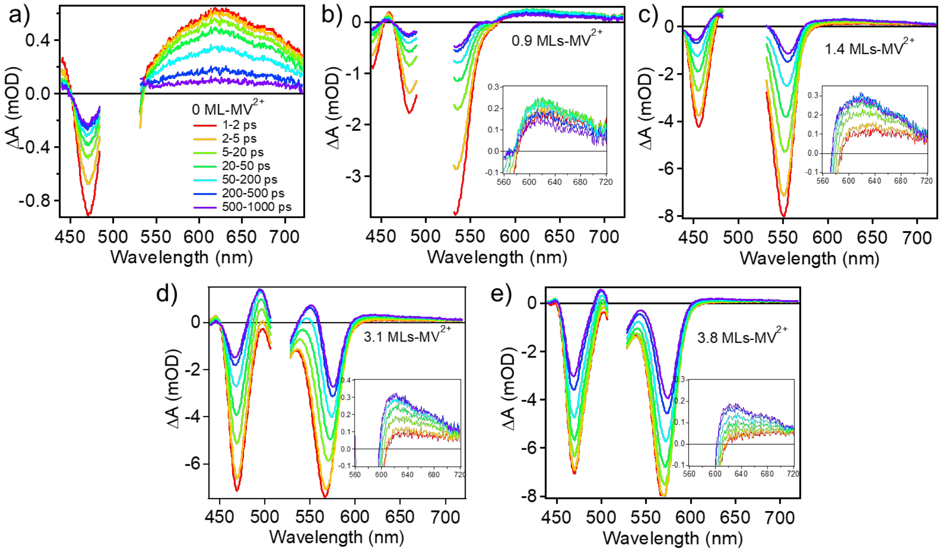

Figure S7: TA spectra of CdSe/CdS QD-MV^{2+} with a). 0, b). 0.9, c). 1.4, d). 3.1 and e). 3.8 monolayers of

CdS shell in time range of 1 ps – 1 ns. The amount of the MV^{2+} was excess compared to that of QDs. The wavelength of the pump pulse was 520 nm. Inset in panel b to panel e: zoomed-in spectra from 560 nm to 720 nm to clearly show the reduced MV^{2+} ($MV^{\bullet+}$) absorption signal as a result of electron transfer. All panels share the same color legend.

In order to determine the rates of electron transfer from QDs to one MV^{2+}, we extracted and fitted the XB kinetics ($1S_h$-$1S_e$ transition of the core) of the QD-MV^{2+} with varying loading amounts of MV^{2+}. As shown in **Figure S8**, with increasing loading amount of MV^{2+}, the XB kinetics show faster decay within the time range of the plots before reaching saturation due to change of apparent electron transfer rate from the change in numbers of adsorbed MV^{2+} on QD surface. The long-lived component in the kinetics in Figure S8 is attributed to free QDs, whose population decreases as more MV^{2+} are added. It is assumed that the numbers of adsorbed MV^{2+} on QD surface follow Poisson distribution (Eq. S1).[9] The apparent rate of electron transfer is proportional to n as shown in Eq. S2. Different from TA spectra of QD with excess MV^{2+} in **Figure S7**, the signal amplitudes of the charge separated state spectra in experiments to determine the k_i are much smaller compared to those of the QD excited state spectra because of the smaller amount of MV^{2+} added to the QD solutions, as indicated by negligible red-shift of the XB peaks and small MV^+ radical signal amplitudes shown in **Figure S9**. Specifically, from Figure S9a to Figure S9e, the fitted average number of adsorbed MV^{2+} (m) is 1.49, 2.00, 0.983, 0.714, and 0.425, respectively, as will be shown in the fitting results below in **Table S6** – **Table S10**. It can be assumed that the charge-separated state signal amplitude is proportional to m. The charge-separated state spectrum in Figure S9a (averaged from 2 to 5 ps) and Figure S9b (averaged from 20 to 100 ps) show less than 1 mOD bleach signal at the corresponding QD XB position. Considering the smaller m number in Figure S9c to Figure S9e, the charge-separated state contribution in these spectra is expected to be even smaller, enabling the direct use of the bleach kinetics to represent the QD XB kinetics. Therefore, the XB kinetics in **Figure S8** are dominated by signal from state filling of $1S_e$ electron level that is proportional to conduction band edge electron populations. Trapped electrons are not involved in these XB kinetics. Also, in the presence of a trapped hole, the band edge electron transfer kinetics should not be affected.[10] Accordingly, the XB kinetics can be derived as:

$$XB(t) = XB(0)(\sum_n e^{-nk_i t} P(n,m))S(t) \quad \text{(S5)}$$

where S(t) is the XB kinetics of QDs without MV^{2+} in the form of two-exponential or three-exponential function. In order to better fit the XB kinetics of the CdSe/CdS QD-MV^{2+}, two components of the electron transfer rates were applied:

$$XB(t) = XB(0)[\sum_n (b_1 e^{-nk_{i1} t} + b_2 e^{-nk_{i2} t})P(n,m)]S(t) \quad \text{(S6)}$$

Eq. S5 and Eq. S6 convoluted with IRF in TA were applied to fit the XB kinetics of CdSe QD-MV^{2+} and CdSe/CdS QD-MV^{2+}, respectively. The necessity of using two electron transfer components may be caused by the heterogeneous binding configurations of MV^{2+} on the QD surface. The summations in the equations were performed until n = 20, considering that the average numbers of adsorbed MV^{2+} are expected to be much smaller than 20 for samples in **Figure S8**, and P(n,m) is negligible when n is larger than 20. We note that the initial amplitude loss of the XB signal compared to pure QDs (without MV^{2+}) is attributed to the fast electron transfer occurring within the IRF. This fast transfer is well accounted for by the above model when the kinetics with different MV^{2+} loading amounts are globally fitted. In addition, Figure S7a and Figure S7b show the decay of the charge-separated state due to charge recombination starting from 5 ps

and 50 ps, respectively. The charge recombination induced XB decay is well separated from the electron transfer induced XB decay and is not involved in the kinetics fitting. The fitting results are shown in **Figure S8** and **Table S6-Table S10**.

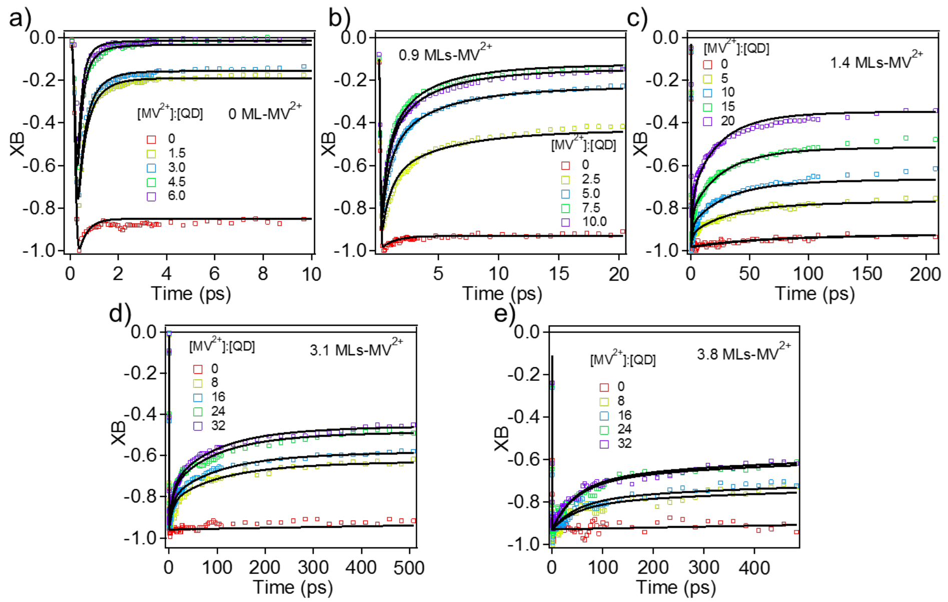

Figure S8: XB kinetics ($1S_h$-$1S_e$ core transition) of CdSe/CdS QD-MV^{2+} with varying amount of MV^{2+} added to QD solution for determination of k_i for electron transfer. a)-e) correspond to CdSe/CdS QDs with 0, 0.9, 1.4, 3.1 and 3.8 monolayers of CdS shell. The wavelength of the pump pulse was 520 nm.

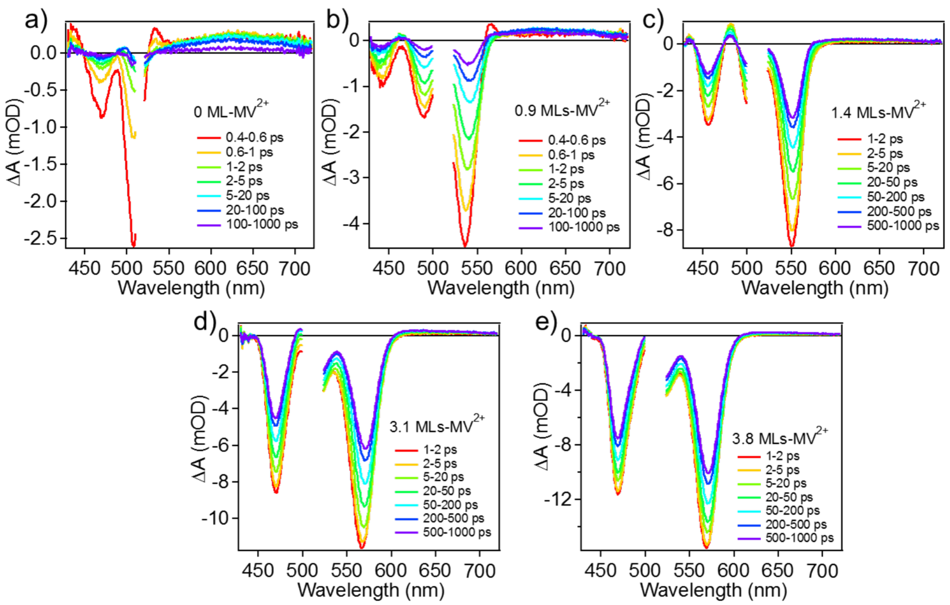

Figure S9: TA spectra of CdSe/CdS QD-MV^{2+} with a). 0, b). 0.9, c). 1.4, d). 3.1 and e). 3.8 monolayers of CdS shell in TA experiments of determination of k_i for electron transfer from QDs. The ratios of MV^{2+} to QDs were 1.5, 10, 20, 32 and 32 from a) to e), corresponding to the average number of adsorbed MV^{2+} of 1.49, 2.00, 0.983, 0.714, and 0.425, respectively, as listed in **Table S6 – Table S10**. The wavelength of the

pump pulse was 520 nm.

$[MV^{2+}]:[QD]$	0	1.5	3.0	4.5	6.0
a_1	0.218 ± 0.016				
τ_1 (ps)	0.370 ± 0.037				
a_2	0.395 ± 0.008				
τ_2 (ns)	13.0 ± 0.4				
a_3	0.389 ± 0.008				
τ_3 (ns)	104 ± 2				
m	0	1.49 ± 0.02	1.68 ± 0.03	3.21 ± 0.10	4.03 ± 0.16
k_i (ps^{-1})	1.46 ± 0.05				
$k_{1/2}$ (ps^{-1})	2.11 ± 0.07				

Table S6: Parameters of global fitting of XB kinetics traces of CdSe QD-MV^{2+} in **Figure S8**. a_i and τ_i (i = 1, 2, 3) are the parameters in three-exponential function to fit the kinetics traces of QD without MV^{2+}.

$[MV^{2+}]:[QD]$	0	2.5	5.0	7.5	10.0
a_1	0.101 ± 0.007				
τ_1 (ps)	1.12 ± 0.21				
a_2	0.649 ± 0.010				
τ_2 (ns)	20.3 ± 0.4				
a_3	0.249 ± 0.010				
τ_3 (ns)	149 ± 7				
m	0	0.760 ± 0.011	1.39 ± 0.02	1.84 ± 0.03	2.00 ± 0.04
b_1	0.530 ± 0.028				
k_{i1} (ps^{-1})	1.15 ± 0.09				
b_2	0.470 ± 0.028				
k_{i2} (ps^{-1})	0.152 ± 0.016				
$k_{1/2}$ (ps^{-1})	0.768 ± 0.077				

Table S7: Parameters of global fitting of XB kinetics traces of CdSe/CdS QD (0.9 monolayers of CdS)-MV^{2+} in **Figure S8**.

$[MV^{2+}]:[QD]$	0	5	10	15	20
a_1	0.047 ± 0.004				
τ_1 (ps)	13.6 ± 3.0				
a_2	0.729 ± 0.007				
τ_2 (ns)	24.9 ± 0.4				
a_3	0.218 ± 0.007				
τ_3 (ns)	223 ± 9				
m	0	0.186 ± 0.005	0.331 ± 0.007	0.589 ± 0.009	0.983 ± 0.015
b_1	0.368 ± 0.014				
k_{i1} (ps^{-1})	0.740 ± 0.070				

b_2	0.632 ± 0.013
k_{i2} (ps^{-1})	0.0314 ± 0.0020
$k_{1/2}$ (ps^{-1})	0.131 ± 0.012

Table S8: Parameters of global fitting of XB kinetics traces of CdSe/CdS QD (1.4 monolayers of CdS)-MV^{2+} in **Figure S8**.

$[MV^{2+}]$:[QD]	0	8	16	24	32
a_1	0.689 ± 0.044				
τ_1 (ns)	18.7 ± 1.0				
a_2	0.311 ± 0.044				
τ_2 (ns)	70.1 ± 7.0				
m	0	0.395 ± 0.015	0.473 ± 0.017	0.655 ± 0.023	0.714 ± 0.025
b_1	0.406 ± 0.037				
k_{i1} (ps^{-1})	0.119 ± 0.020				
b_2	0.594 ± 0.036				
k_{i2} (ps^{-1})	0.0844 ± 0.0014				
$k_{1/2}$ (ps^{-1})	0.141 ± 0.014				

Table S9: Parameters of global fitting of XB kinetics traces of CdSe/CdS QD (3.1 monolayers of CdS)-MV^{2+} in **Figure S8**.

$[MV^{2+}]$:[QD]	0	8	16	24	32
a_1	0.531 ± 0.063				
τ_1 (ns)	14.4 ± 1.2				
a_2	0.469 ± 0.063				
τ_2 (ns)	47.0 ± 3.9				
m	0	0.201 ± 0.092	0.237 ± 0.109	0.407 ± 0.198	0.425 ± 0.208
b_1	0.684 ± 0.130				
k_{i1} (ps^{-1})	0.0187 ± 0.0082				
b_2	0.316 ± 0.130				
k_{i2} (ps^{-1})	$(2.615 \pm 0.918) \times 10^{-3}$				
$k_{1/2}$ (ps^{-1})	0.0171 ± 0.0078				

Table S10: Parameters of global fitting of XB kinetics traces of CdSe/CdS QD (3.8 monolayers of CdS)-MV^{2+} in **Figure S8**.

Different from the triplet energy transfer, the electron transfer is better described by a two-exponential decay function rather than a single exponential decay function. This complexity is due to the heterogeneous nature of the electron transfer process.[11] For such a multiexponential decay process, the amplitude weighted average lifetime or the half lifetime is usually reported. Here, we choose to use the rate constant corresponding to the half lifetime ($k_{1/2}$) for the calculation of coupling strength in Eq. 3, Eq. 4 and Eq. 5., rather than the amplitude weighted average rate constant (k_{avg}). This is because k_{avg} sensitively depends on fast exponential decay components, while $k_{1/2}$ offers a better representation of the whole multiexponential decay.

$k_{1/2}$ of electron transfer is calculated from the fitted b_1, b_2, k_{i1}, and k_{i2} in Eq. S6. Specifically, the intrinsic electron transfer induced decay of the band edge electron population can be written as Eq. S6-1, as embedded in Eq. S6:

$$f(t) = b_1 e^{-k_{i1}t} + b_2 e^{-k_{i2}t} \qquad \text{(S6-1)}$$

The half lifetime ($\tau_{1/2}$) of the electron transfer induced decay is then found by setting $f(t) = 1/2$, and $k_{1/2}$ is calculated as $k_{1/2} = \tau_{1/2}{}^{-1}$. $k_{1/2}$ of triplet energy transfer and hole transfer are obtained in the same way, with the results shown in **Table S1** – **Table S11**.

SI9. Determination of Rates of Hole Transfer from CdSe/CdS QDs to Phenothiazine (PTZ)

Shell thickness dependent rates of hole transfer from CdSe/CdS QDs were studied with PTZ as the hole acceptor. As shown in **Figure S10**, the TA spectra of CdSe/CdS QDs adsorbed with excess PTZ show formation of derivative-like features due to Stark effect from charge separation and show no faster decay of $1S_h$-$1S_e$ XB in range of 500 nm to 600 nm, suggesting hole transfer from QDs to PTZ considering that XB in CdSe/CdS QDs is mainly attributed to state filling of 1S electron level.[7] One should note that the PTZ^+ radical signal at around 520 nm overlaps with the QD excited state spectra and charge separated state spectra and cannot be well resolved in TA spectra because of its small amplitude.

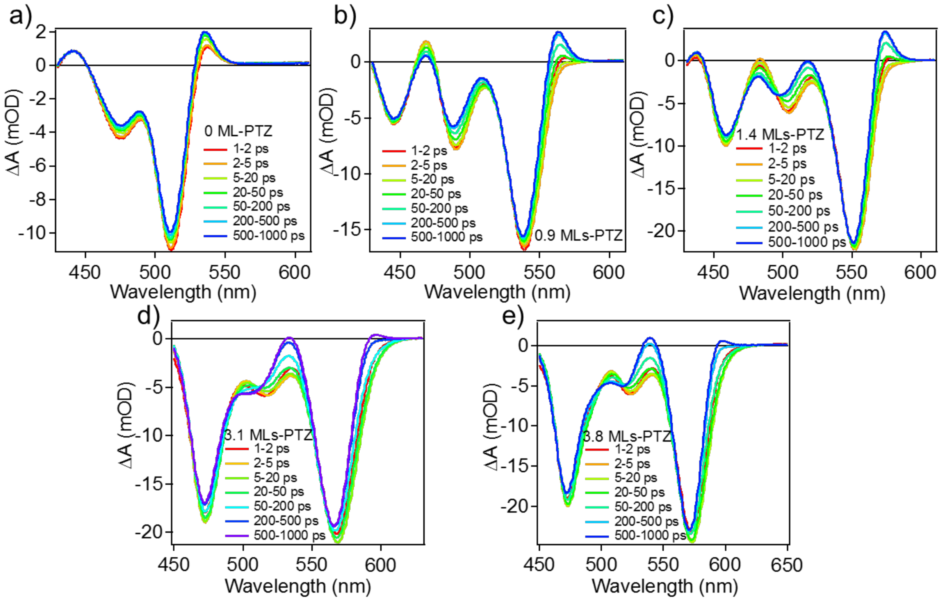

Figure S10: TA spectra of CdSe/CdS QD-PTZ with a). 0, b). 0.9, c). 1.4, d). 3.1 and e). 3.8 monolayers of CdS shell in time range of 1 ps – 1 ns. The amount of the PTZ was excess compared to that of QDs. The wavelength of the pump pulse was 400 nm.

Because XB signal of the core-shell QDs in TA mainly consists of state filling of conduction band edge electron, we turned to TRPL measurement when determining rates of hole transfer from QDs to one PTZ (k_i). The excitation wavelength was set as 460 nm due to limitation of the laser for TRPL. Despite the inevitable excitation of T band transition by 460 nm, the hole generated by excitation will either be trapped

or relax to $1S_h$ level of the QD in much shorter time compared to the instrument response of the TRPL measurement. Because only band edge emission was monitored in TRPL, holes in deep trap states would not be detected in TRPL. Therefore, the TRPL kinetics mainly show the dynamics of hole transfer from $1S_h$ level to PTZ. In order to determine the k_i of hole transfer, we measured the TRPL kinetics with varying loading amounts of PTZ and extracted the k_i of hole transfer from global fitting, which is similar to the determination of k_i for triplet energy transfer and electron transfer in the study. Because of the relatively fast hole transfer, the detection time window for TRPL was set as 50 ns, with FWHM of 62 ps for the Gaussian IRF. With the assumption of Poisson distribution of the numbers of adsorbed PTZ, TRPL kinetics of QD-PTZ can be derived in a similar manner to Eq. S4. The expression is:

$$N(t,m) = \sum_j (N(0)(\sum_n (c_1 e^{-nk_{i1}(t+jT_R)} + c_2 e^{-nk_{i2}(t+jT_R)} + c_3 e^{-nk_{i3}(t+jT_R)}) P(n,m)) S(t+jT_R)) \quad (S7)$$

where S(t) is the TRPL kinetics of the free QD without PTZ, and T_R is the repetition time of the pulse.[7] The summations of j and n were performed until j = 10 and n = 20. Note that the hole transfer induced decay is best fit by a three-exponential decay function rather than a single-exponential decay function, probably due to the heterogeneous binding configurations of PTZ on the QD surface. The rate constant corresponding to the half lifetimes ($k_{1/2}$) of the hole transfer induced decay function in different samples are used to study the shell thickness dependence in the main text, similar to the electron transfer part. The fitting result is shown in **Figure S11** and **Table S11**.

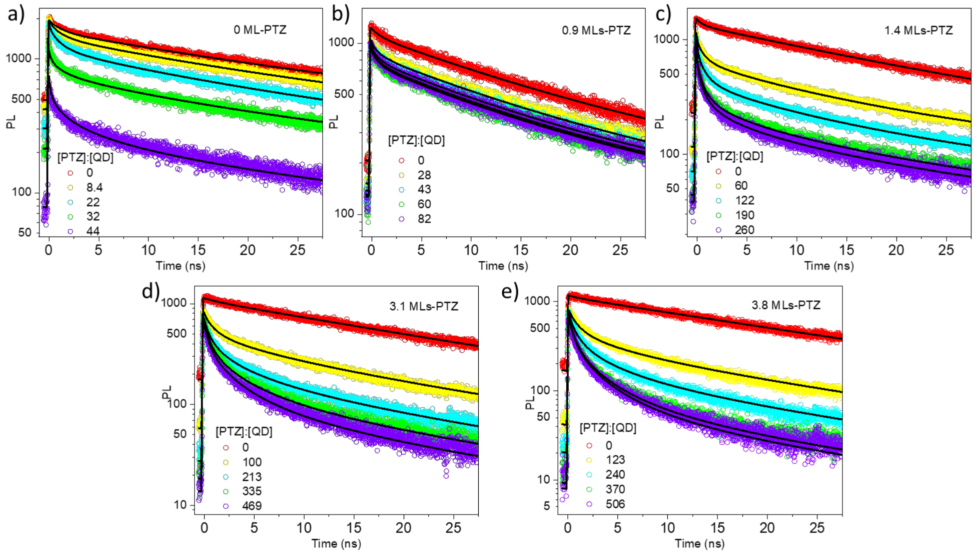

Figure S11: TRPL kinetics at band edge emission peaks of CdSe/CdS QDs-PTZ with a). 0, b). 0.9, c). 1.4, d). 3.1 and e). 3.8 monolayers of CdS shell and varying PTZ to QD ratios. The detection time window was 50 ns. The global fitting curves of the kinetics traces are shown as black solid lines.

Monolayers of CdS shell	0	0.9	1.4	3.1	3.8
m_0	0	0	0	0	0
m_1	0.338 ± 0.034	0.309 ± 0.003	0.981 ± 0.034	1.111 ± 0.002	1.399 ± 0.003

m_2	0.587 ± 0.038	0.407 ± 0.003	1.34 ± 0.05	1.856 ± 0.005	2.12 ± 0.01
m_3	0.838 ± 0.002	0.489 ± 0.004	1.85 ± 0.03	2.262 ± 0.007	2.92 ± 0.01
m_4	1.84 ± 0.01	0.460 ± 0.004	1.99 ± 0.03	2.549 ± 0.008	3.05 ± 0.01
c_1	0.540 ± 0.020	0.556 ± 0.036	0.483 ± 0.015	0.228 ± 0.004	0.278 ± 0.003
k_{i1} (ns^{-1})	38.5 ± 3.86	12.9 ± 1.8	9.94 ± 0.52	30.0 ± 2.2	29.4 ± 1.7
c_2	0.271 ± 0.019	0.238 ± 0.033	0.383 ± 0.015	0.468 ± 0.005	0.455 ± 0.005
k_{i2} (ns^{-1})	3.30 ± 0.36	1.53 ± 0.30	1.25 ± 0.09	0.902 ± 0.025	0.672 ± 0.018
c_3	0.189 ± 0.028	0.206 ± 0.048	0.134 ± 0.021	0.303 ± 0.006	0.267 ± 0.006
k_{i3} (ns^{-1})	0.119 ± 0.007	0.084 ± 0.012	0.154 ± 0.023	0.106 ± 0.003	0.092 ± 0.003
$k_{1/2}$ (ns^{-1})	20.2 ± 3.6	7.63 ± 1.88	5.15 ± 0.68	1.21 ± 0.04	1.15 ± 0.05

Table S11: Parameters of global fitting of TRPL kinetics traces of CdSe/CdS QD-PTZ in **Figure S11**. The parameters of S(t) are the same as those in fitting of TRPL kinetics traces of CdSe/CdS QD-ACA and thus are not shown in the table. m_i (i=0, 1, 2, 3, 4) refers to average numbers of adsorbed PTZ on QD surface with increasing concentrations of PTZ added to QD solution.

The fitted intrinsic rate constants for TET, electron transfer, and hole transfer are summarized in **Figure S12**. Note that the rate constant corresponding to the half lifetime of each transfer process was chosen for the discussion in the main text and SI. The rate constants overall decay exponentially with increasing shell thickness *r*. The trend was fit with:

$$k = Ae^{-\beta_k r} \quad or \quad ln(k) = a - \beta_k r \qquad (S8)$$

where β_k is the rate decay constant. The $\beta_{k_{ET}}$ is comparable with literature,[12, 13] as discussed in the main text. Note that the exponential fitting of the size-dependent rate constants in Figure S12 is only phenomenological. The rate constants were used to extract the electronic coupling using Eq. 3 – Eq. 5 in the main text.

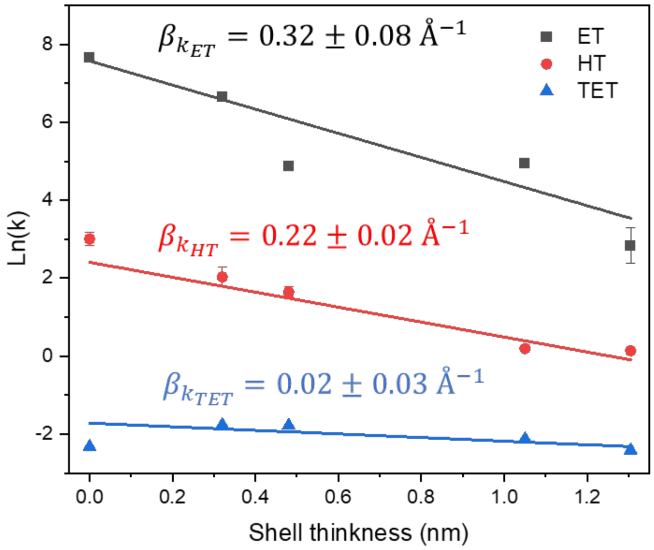

Figure S12. Intrinsic rate constants for electron transfer (ET) (black squares), hole transfer (HT) (red dots), and TET (blue triangles). The solid lines are fittings to equation S8. The related rate decay constants are noted.

SI10. Core/shell QDs wave functions and excitation energies

The electronic structure of CdSe/CdS core/shell QDs was modeled using the effective mass approximation with the discrete variable representation (DVR) method.[14, 15] **Table S12** shows the effective masses and the valence and conduction band potentials used for the QDs.[16] The computed energies and wave functions for the 1s conduction and valance band states were computed with the DVR method. The computed excitation energies as a function of shell thickness are consistent with the measured band edge excitation energies found in the experimental UV-vis spectra (Table S13). The wave functions obtained from the DVR calculations are grid based. For further analysis of electronic interactions, the 1s wave functions were modeled using the radial wave function $\psi_{1s} = \frac{1}{\sqrt{4\pi}} R(r)$, where R(r) is the zeroth-order spherical Bessel function ($\frac{\sin \kappa r}{\kappa r}$) and spherical Hankel function of the first kind ($\frac{\exp \kappa r}{\kappa r}$), when the energy E is lower or higher than the potential V, respectively. Here, $\kappa = \sqrt{\frac{2m^*|V-E|}{\hbar^2}}$ where $\hbar$ is Planck's constant divided by 2p, V is the potential in a given region, and m^* is the effective mass (see **Table S12**). The coefficients of the Bessel and Hankel functions in each region are determined by the continuity conditions at the core/shell and shell/solvent boundaries, and by wave function normalization. These analytical wave functions replicate the wave functions computed using the DVR analysis (**Figure S13**). The electron delocalizes over the entire core/shell QD, while the hole localizes mainly in the core region, due to the high

hole potential barrier in the CdS shell region. The change in the shell thickness weakly influences the hole delocalization. This finding is consistent with the semi-type II semiconductor band alignment for CdSe/CdS core/shell QD structures.[16]

	CdSe	CdS	hexane
m_e^* / m_0	0.13	0.21	1
m_h^* / m_0	0.45	0.80	1
V_e / eV	-4.0	-3.78	0
V_h / eV	-5.7	-6.29	-8.4

Table S12: effective masses and potentials for the CdSe/CdS core/shell DVR analysis.[16]

Shell thickness (nm)	$E_{1S,ele}(eV)$	$E_{1S,hole}(eV)$	$E_{1S,ele-1S,hole}$ (eV)	$E_{ex,UV-vis}$ (eV)
0	-3.42	-5.97	2.54	2.42
0.32	-3.57	-5.91	2.34	2.30
0.48	-3.60	-5.90	2.30	2.25
1.05	-3.68	-5.90	2.22	2.19
1.3	-3.70	-5.90	2.20	2.18

Table S13: computed electron 1s state energy, hole 1s state energy and excitation energy of CdSe/CdS QDs at 0, 0.32, 0.48, 1.05 and 1.3 nm shell thickness with experimental excitation energy.

Shell thickness (nm)	$1S_e$ (eV)	$1P_e$ (eV)	$1D_e$ (eV)	$2S_e$ (eV)
0	-3.42	-2.70	-1.76	-0.97
0.32	-3.56	-3.04	-2.40	-1.78
0.48	-3.60	-3.14	-2.59	-2.09
1.05	-3.68	-3.37	-3.05	-2.86
1.3	-3.70	-3.43	-3.18	-3.05

Table S14: Computed electron 1S, 1P, 1D and 2S state energy for CdSe/CdS QDs

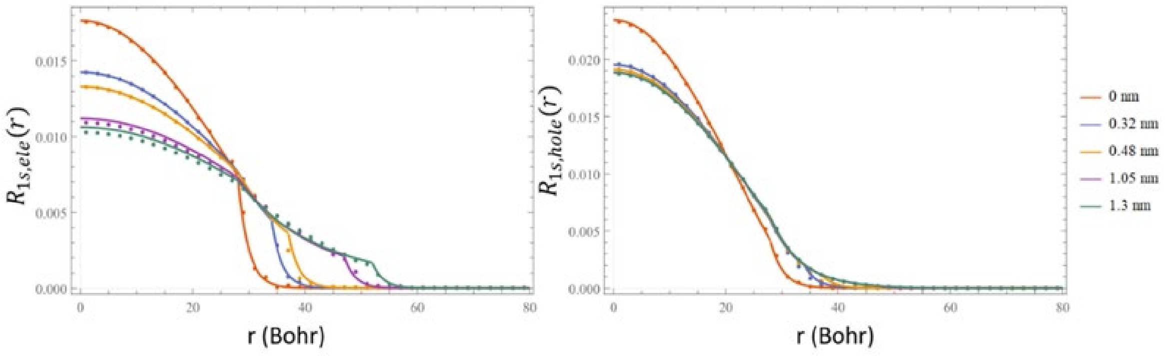

Figure 13. The radial 1S (A) electron and (B) hole wave function of CdSe/CdS QDs at 0, 0.32, 0.48, 1.05 and 1.3 nm shell thickness. Dots represent the numerical wave function computed with DVR method and the solid lines represent the fitted wave function.

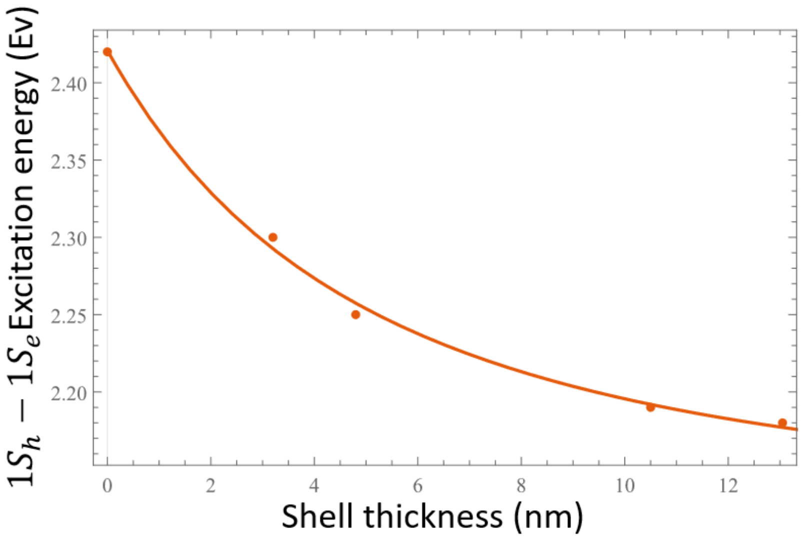

Figure S14. The experimental and fit CdSe/CdS core/shell QDs $\mathbf{1S_h}$**-1S_e** excitation energy as the function of shell thickness. The excitation energy is fit as $\mathbf{2.12 + \frac{29.5}{(9.9+d)^2}}$ where d is the shell thickness.

SI11. Calculation of Reorganization Energies of the Acceptors for Electron/hole Transfer

The total reorganization energy is the sum of the two components, $\lambda = \lambda_{QD} + \lambda_{ACC}$. The acceptor (ACC) component of reorganization energy λ_{ACC} is defined as the difference between the energy of ACC^* at the equilibrium geometry of ACC, i.e., electron/hole/triplet-exciton acceptor's initial state, and the energy of ACC^* at the equilibrium geometry of ACC^*, its final state. For this we use the B3LYP functional[17, 18] with a 6-31++G(d,p) basis as implemented in Gaussian 09.[19] The calculated values for each of the acceptor's reorganization energy are λ_{MV} = 300, λ_{PTZ} = 87 , λ_{ACA} = 317 meV. **Tables S14-S16** summarize the geometries of the three acceptor molecules before and after ET/HT/TET. Note that different calculation methods give the reorganization energy of ACA between 0.2 and 0.4 eV. We chose λ_{ACA} = 0.22 eV from literature[20] for the discussion in the main text because it fits the experimental data best.

Table S14a. $MV^+@MV^{2+}$ Cartesian coordinates in Å before ET. E(ROB3LYP) = -575.026131509 E_h

Atom	X	Y	Z
C	0.743468	0.005678	0.003632
C	1.469384	1.130968	-0.415856
C	2.854860	1.102843	-0.404640
C	2.850409	-1.093547	0.429638

C	1.466439	-1.120429	0.432076
C	-0.743468	0.005678	-0.003634
C	-1.469383	1.130968	0.415854
C	-2.854859	1.102843	0.404639
C	-2.850409	-1.093547	-0.429639
C	-1.466439	-1.120428	-0.432078
H	0.977239	2.027024	-0.776499
H	3.453386	1.944645	-0.729893
H	3.448700	-1.935504	0.757262
H	0.970047	-2.014364	0.792483
H	-0.977238	2.027024	0.776497
H	-3.453386	1.944645	0.729893
H	-3.448701	-1.935504	-0.757262
H	-0.970048	-2.014364	-0.792486
C	5.012661	-0.021137	-0.019195
H	5.380987	-0.457938	0.908846
H	5.336150	-0.619355	-0.873295
H	5.381976	0.997920	-0.116656
C	-5.012661	-0.021138	0.019199
H	-5.336148	-0.619350	0.873304
H	-5.381976	0.997921	0.116654
H	-5.380989	-0.457945	-0.908838
N	3.524877	0.004474	0.012384
N	-3.524877	0.004474	-0.012384

Table S14b. MV^{+} Cartesian coordinates in Å after ET, E(ROB3LYP) = -575.037331083 E_h

Atom	X	Y	Z
C	0.716380	0.001480	0.000196
C	1.487728	1.204486	-0.049535
C	2.842913	1.178742	-0.046558
C	2.843690	-1.172368	0.054182
C	1.489839	-1.200788	0.051243
C	-0.716386	0.001480	-0.000195
C	-1.487732	1.204485	0.049535
C	-2.842911	1.178741	0.046558
C	-2.843687	-1.172367	-0.054182
C	-1.489843	-1.200787	-0.051243
H	1.017674	2.177402	-0.103676
H	3.435480	2.083664	-0.090655
H	3.440524	-2.075120	0.098466
H	1.020318	-2.174232	0.103582

H	-1.017680	2.177401	0.103677
H	-3.435478	2.083662	0.090654
H	-3.440521	-2.075118	-0.098466
H	-1.020324	-2.174231	-0.103581
C	5.007470	-0.013092	-0.007486
H	5.380579	-0.574470	0.852499
H	5.368478	-0.475417	-0.930321
H	5.377827	1.009904	0.049067
C	-5.007465	-0.013092	0.007486
H	-5.368473	-0.475403	0.930329
H	-5.377823	1.009903	-0.049083
H	-5.380575	-0.574483	-0.852490
N	3.538694	0.005358	0.007830
N	-3.538690	0.005358	-0.007831

Table S15a. PTZ^+@PTZ Cartesian coordinates in Å before HT, E(UB3LYP) = -915.421871567 E_h

Atom	X	Y	Z
C	0.000000	3.817857	0.391670
C	0.000000	2.672027	1.193331
C	0.000000	1.394353	0.628365
C	0.000000	1.250683	-0.772160
C	0.000000	2.406081	-1.569244
C	0.000000	3.678765	-0.996550
C	-0.000000	-1.250683	-0.772160
C	-0.000000	-1.394353	0.628365
C	-0.000000	-2.672027	1.193331
H	0.000000	-2.770529	2.275654
C	-0.000000	-3.817857	0.391670
C	-0.000000	-3.678765	-0.996550
C	-0.000000	-2.406081	-1.569244
H	0.000000	4.800436	0.852429
H	0.000000	2.770529	2.275654
H	0.000000	2.298266	-2.651796
H	0.000000	4.553696	-1.639595
H	-0.000000	0.000000	-2.397464
H	-0.000000	-4.800436	0.852429
H	-0.000000	-4.553696	-1.639595
H	-0.000000	-2.298266	-2.651796
N	-0.000000	0.000000	-1.388783
S	0.000000	-0.000000	1.746286

Table S15b. PTZ^+ Cartesian coordinates in Å after HT, E(UB3LYP) = -915.425032857 E_h

Atom	X	Y	Z
C	-3.783009	-0.404636	-0.000279
C	-2.657286	-1.213743	-0.000095
C	-1.373207	-0.638632	0.000112
C	-1.238924	0.775079	0.000185
C	-2.396340	1.583603	-0.000049
C	-3.649450	0.999896	-0.000298
C	1.238935	0.775043	0.000127
C	1.373165	-0.638671	0.000057
C	2.657310	-1.213749	-0.000063
H	2.759666	-2.294724	-0.000002
C	3.782997	-0.404657	-0.000229
C	3.649459	0.999924	-0.000325
C	2.396361	1.583586	-0.000100
H	-4.769492	-0.855750	-0.000387
H	-2.759731	-2.294708	-0.000093
H	-2.291053	2.665147	-0.000087
H	-4.534077	1.627870	-0.000565
H	0.000009	2.392748	0.001003
H	4.769519	-0.855694	-0.000283
H	4.534136	1.627816	-0.000584
H	2.290951	2.665120	-0.000122
N	0.000028	1.378001	0.000600
S	-0.000012	-1.721381	0.000167

Table S16a. ACA(T1@S0) Cartesian coordinates in Å before TET, E(UB3LYP) = -728.050532890 E_h

Atom	X	Y	Z
C	-2.205387	-4.092461	-0.027470
C	-0.835814	-4.065349	-0.030656
C	-0.121679	-2.824678	-0.025901
C	-0.855935	-1.578745	-0.033010
C	-2.287924	-1.658404	-0.019635
C	-2.935132	-2.868859	-0.016209
C	1.276506	-2.800315	-0.009757
C	-0.132523	-0.358799	-0.005801
C	1.286092	-0.343688	0.040870
C	1.997309	-1.602285	0.021418
C	3.428700	-1.604136	0.035922
H	3.942243	-2.561989	0.021018
C	4.135583	-0.431114	0.065496

C	3.440054	0.812304	0.082926
C	2.068154	0.858178	0.072360
H	1.820177	-3.742532	-0.016300
H	-2.737830	-5.038951	-0.028244
H	-0.262256	-4.988654	-0.030586
H	-2.870413	-0.746367	-0.018042
H	-4.021015	-2.894855	-0.004231
H	5.221492	-0.442757	0.076203
H	4.005273	1.739555	0.109354
H	1.565000	1.815752	0.107022
C	-0.851049	0.953121	-0.003206
O	-0.649551	1.868650	0.772466
O	-1.769959	1.067117	-0.999316
H	-2.166058	1.951002	-0.906201

Table S16b. ACA(T1) Cartesian coordinates in Å after TET, E(UB3LYP) = -728.062168929 E_h

Atom	X	Y	Z
C	-2.244347	-4.086373	-0.008574
C	-0.841751	-4.041757	-0.091246
C	-0.145081	-2.821816	-0.085494
C	-0.880339	-1.585453	-0.032923
C	-2.276430	-1.663617	0.077082
C	-2.956254	-2.900439	0.088051
C	1.279182	-2.795327	-0.106834
C	-0.138740	-0.324521	-0.042448
C	1.321335	-0.320108	0.054943
C	2.016936	-1.580199	-0.007126
C	3.420547	-1.598016	0.030987
H	3.930761	-2.556321	-0.021246
C	4.168568	-0.411055	0.131621
C	3.504867	0.803727	0.195329
C	2.093647	0.847659	0.155923
H	1.821222	-3.735760	-0.155746
H	-2.757879	-5.043240	-0.013459
H	-0.273478	-4.966133	-0.153803
H	-2.860985	-0.756636	0.145102
H	-4.038967	-2.909806	0.167037
H	5.253352	-0.454441	0.159593
H	4.059760	1.733387	0.276344
H	1.597822	1.806263	0.219765
C	-0.842282	0.970193	-0.160891

O	-0.515589	2.018530	0.379765
O	-1.929757	0.942885	-0.986725
H	-2.282065	1.849112	-0.984537

REFERENCE

(1) Chen, O.; Zhao, J.; Chauhan, V. P.; Cui, J.; Wong, C.; Harris, D. K.; Wei, H.; Han, H.-S.; Fukumura, D.; Jain, R. K.; et al. Compact high-quality CdSe–CdS core–shell nanocrystals with narrow emission linewidths and suppressed blinking. *Nature Materials* **2013**, *12*, 445, Article. DOI: 10.1038/nmat3539 https://www.nature.com/articles/nmat3539#supplementary-information.

(2) Hanifi, D. A.; Bronstein, N. D.; Koscher, B. A.; Nett, Z.; Swabeck, J. K.; Takano, K.; Schwartzberg, A. M.; Maserati, L.; Vandewal, K.; van de Burgt, Y.; et al. Redefining near-unity luminescence in quantum dots with photothermal threshold quantum yield. *Science* **2019**, *363* (6432), 1199-1202. DOI: 10.1126/science.aat3803.

(3) Zhu, H.; Song, N.; Rodríguez-Córdoba, W.; Lian, T. Wave Function Engineering for Efficient Extraction of up to Nineteen Electrons from One CdSe/CdS Quasi-Type II Quantum Dot. *Journal of the American Chemical Society* **2012**, *134* (9), 4250-4257. DOI: 10.1021/ja210312s.

(4) Grimaldi, G.; Geuchies, J. J.; van der Stam, W.; du Fossé, I.; Brynjarsson, B.; Kirkwood, N.; Kinge, S.; Siebbeles, L. D. A.; Houtepen, A. J. Spectroscopic Evidence for the Contribution of Holes to the Bleach of Cd-Chalcogenide Quantum Dots. *Nano Letters* **2019**. DOI: 10.1021/acs.nanolett.9b00164.

(5) Jin, T.; Lian, T. Trap state mediated triplet energy transfer from CdSe quantum dots to molecular acceptors. *The Journal of Chemical Physics* **2020**, *153* (7), 074703. DOI: 10.1063/5.0022061 (acccessed 2020/09/24).

(6) Mongin, C.; Garakyaraghi, S.; Razgoniaeva, N.; Zamkov, M.; Castellano, F. N. Direct observation of triplet energy transfer from semiconductor nanocrystals. *Science* **2016**, *351* (6271), 369-372. DOI: 10.1126/science.aad6378.

(7) Huang, J. E.; Huang, Z. Q.; Jin, S. Y.; Lian, T. Q. Exciton Dissociation in CdSe Quantum Dots by Hole Transfer to Phenothiazine. *J Phys Chem C* **2008**, *112* (49), 19734-19738. DOI: 10.1021/jp808291u.

(8) Xu, Z.; Jin, T.; Huang, Y.; Mulla, K.; Evangelista, F. A.; Egap, E.; Lian, T. Direct triplet sensitization of oligothiophene by quantum dots. *Chem. Sci.* **2019**, *10* (24), 6120-6124, 10.1039/C9SC01648A. DOI: 10.1039/c9sc01648a.

(9) Chen, J.; Wu, K.; Rudshteyn, B.; Jia, Y.; Ding, W.; Xie, Z. X.; Batista, V. S.; Lian, T. Ultrafast Photoinduced Interfacial Proton Coupled Electron Transfer from CdSe Quantum Dots to 4,4'-Bipyridine. *J. Am. Chem. Soc.* **2016**, *138* (3), 884-892. DOI: 10.1021/jacs.5b10354.

(10) He, S.; Ni, A.; Gebre, S. T.; Hang, R.; McBride, J. R.; Kaledin, A. L.; Yang, W.; Lian, T. Doping of Colloidal Nanocrystals for Optimizing Interfacial Charge Transfer: A Double-Edged Sword. *Journal of the American Chemical Society* **2024**. DOI: 10.1021/jacs.4c06110.

(11) Wu, K.; Du, Y.; Tang, H.; Chen, Z.; Lian, T. Efficient Extraction of Trapped Holes from Colloidal CdS Nanorods. *J Am Chem Soc* **2015**, *137* (32), 10224-10230. DOI: 10.1021/jacs.5b04564.

(12) Dworak, L.; Matylitsky, V. V.; Breus, V. V.; Braun, M.; Basché, T.; Wachtveitl, J. Ultrafast Charge Separation at the CdSe/CdS Core/Shell Quantum Dot/Methylviologen Interface: Implications for Nanocrystal Solar Cells. *The Journal of Physical Chemistry C* **2011**, *115* (10), 3949-3955. DOI: 10.1021/jp111574w.

(13) Zeng, P.; Kirkwood, N.; Mulvaney, P.; Boldt, K.; Smith, T. A. Shell effects on hole-coupled electron transfer dynamics from CdSe/CdS quantum dots to methyl viologen. *Nanoscale* **2016**, *8* (19), 10380-10387. DOI: 10.1039/c6nr00168h.

(14) Zhu, H.; Yang, Y.; Hyeon-Deuk, K.; Califano, M.; Song, N.; Wang, Y.; Zhang, W.; Prezhdo, O. V.; Lian, T. Auger-assisted electron transfer from photoexcited semiconductor quantum dots. *Nano Lett* **2014**, *14* (3), 1263-1269. DOI: 10.1021/nl4041687.

(15) Kaledin, A. L.; Lian, T.; Hill, C. L.; Musaev, D. G. An Infinite Order Discrete Variable Representation of an Effective Mass Hamiltonian: Application to Exciton Wave Functions in Quantum Confined Nanostructures. *Journal of Chemical Theory and Computation* **2014**, *10* (8), 3409-3416. DOI: 10.1021/ct500280j.

(16) Zhu, H.; Song, N.; Rodriguez-Cordoba, W.; Lian, T. Wave Function Engineering for Efficient Extraction of up to Nineteen Electrons from One CdSe/CdS Quasi-Type II Quantum Dot. *J Am Chem Soc* **2012**, *134* (9), 4250-4257. DOI: 10.1021/ja210312s.

(17) Becke, A. D. Density-functional exchange-energy approximation with correct asymptotic behavior. *Physical Review A* **1988**, *38* (6), 3098-3100. DOI: 10.1103/PhysRevA.38.3098.

(18) Lee, C.; Yang, W.; Parr, R. G. Development of the Colle-Salvetti correlation-energy formula into a functional of the electron density. *Physical Review B* **1988**, *37* (2), 785-789. DOI: 10.1103/PhysRevB.37.785.

(19) Frisch, M. J. T., G. W.; Schlegel, H. B.; Scuseria, G. E.; Robb, M. A.; Cheeseman, J. R.; Scalmani, G.; Barone, V.; Mennucci, B.; Petersson, G. A.; Nakatsuji, H.; Caricato, M.; Li, X.; Hratchian, H. P.; Izmaylov, A. F.; Bloino, J.; Zheng, G.; Sonnenberg, J. L.; Hada, M.; Ehara, M.; Toyota, K.; Fukuda, R.; Hasegawa, J.; Ishida, M.; Nakajima, T.; Honda, Y.; Kitao, O.; Nakai, H.; Vreven, T.; Montgomery, J. A., Jr.; Peralta, J. E.; Ogliaro, F.; Bearpark, M.; Heyd, J. J.; Brothers, E.; Kudin, K. N.; Staroverov, V. N.; Kobayashi, R.; Normand, J.; Raghavachari, K.; Rendell, A.; Burant, J. C.; Iyengar, S. S.; Tomasi, J.; Cossi, M.; Rega, N.; Millam, M. J.; Klene, M.; Knox, J. E.; Cross, J. B.; Bakken, V.; Adamo, C.; Jaramillo, J.; Gomperts, R.; Stratmann, R. E.; Yazyev, O.; Austin, A. J.; Cammi, R.; Pomelli, C.; Ochterski, J. W.; Martin, R. L.; Morokuma, K.; Zakrzewski, V. G.; Voth, G. A.; Salvador, P.; Dannenberg, J. J.; Dapprich, S.; Daniels, A. D.; Farkas, Ö.; Foresman, J. B.; Ortiz, J. V.; Cioslowski, J.; Fox, D. J. Gaussian 09, Revision D.01. *Gaussian, Inc., Wallingford CT* **2009**.

(20) Lai, R.; Liu, Y.; Luo, X.; Chen, L.; Han, Y.; Lv, M.; Liang, G.; Chen, J.; Zhang, C.; Di, D.; et al. Shallow distance-dependent triplet energy migration mediated by endothermic charge-transfer. *Nature Communications* **2021**, *12* (1). DOI: 10.1038/s41467-021-21561-1.
